# Supplementary material for: Utilization of Water-Soluble Aminoethylamino–β–Cyclodextrin in the Pfitzinger Reaction—Catalyzed to the Synthesis of Diversely Functionalized Quinaldine
Source: Polymers (Basel). 2020 Feb 9;12(2):393. doi: 10.3390/polym12020393 (PMC7077625; doi:10.3390/polym12020393)

polymers

Supplementary Materials

1. Characterization techniques and data for AEA-β-CD

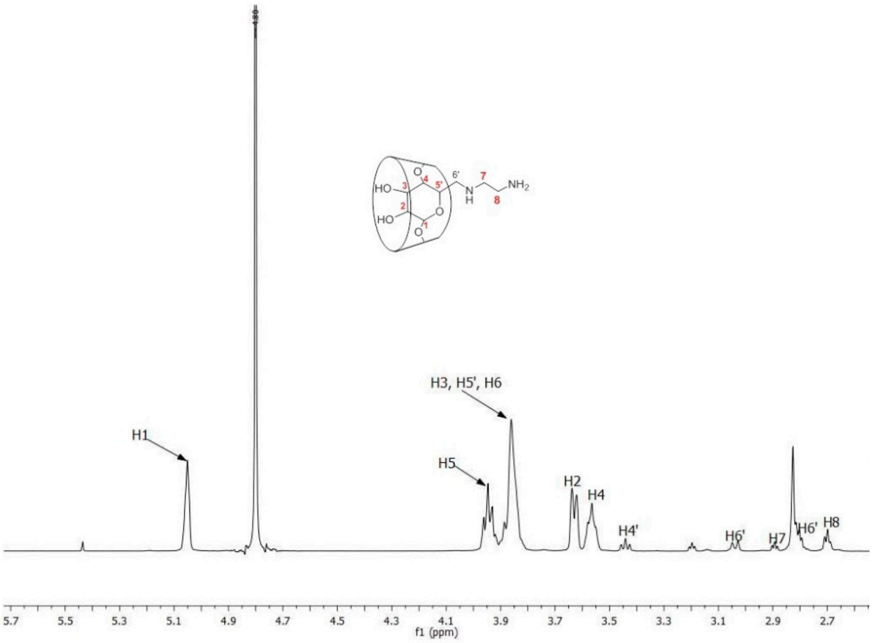

Figure S1. ^{1}H spectra of AEA-β-CD measured in D_2O.

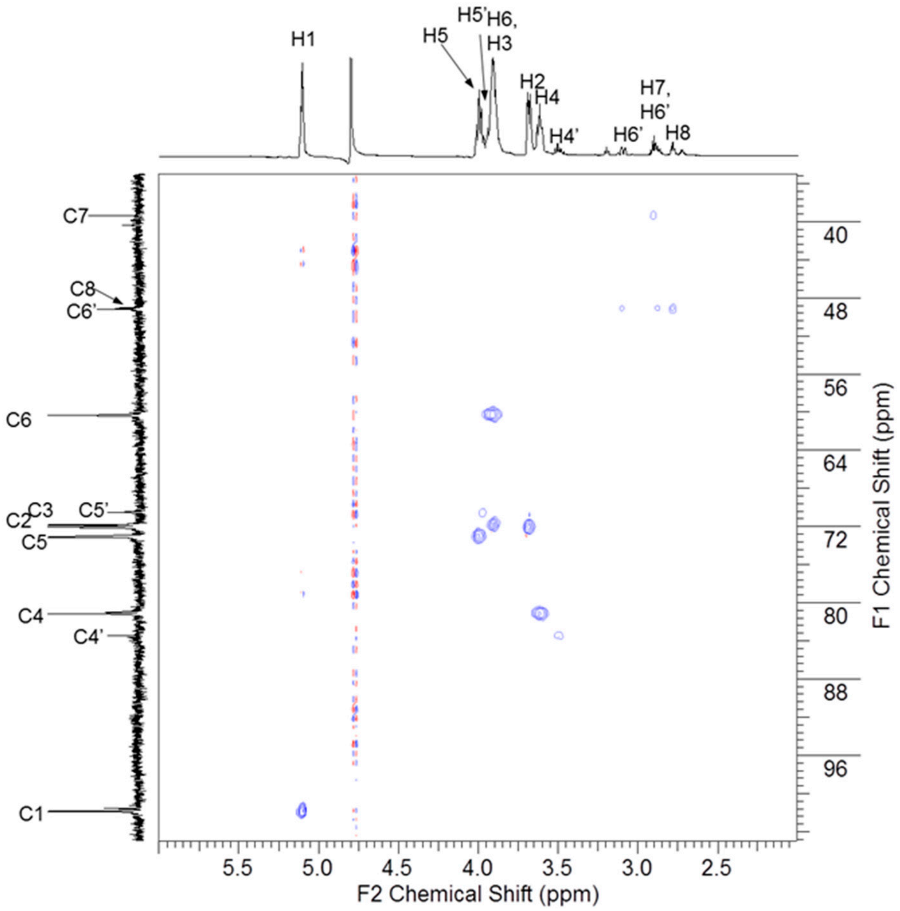

Figure S2. HSQC NMR spectrum

2. Recovered yield of the AEA-β-CD

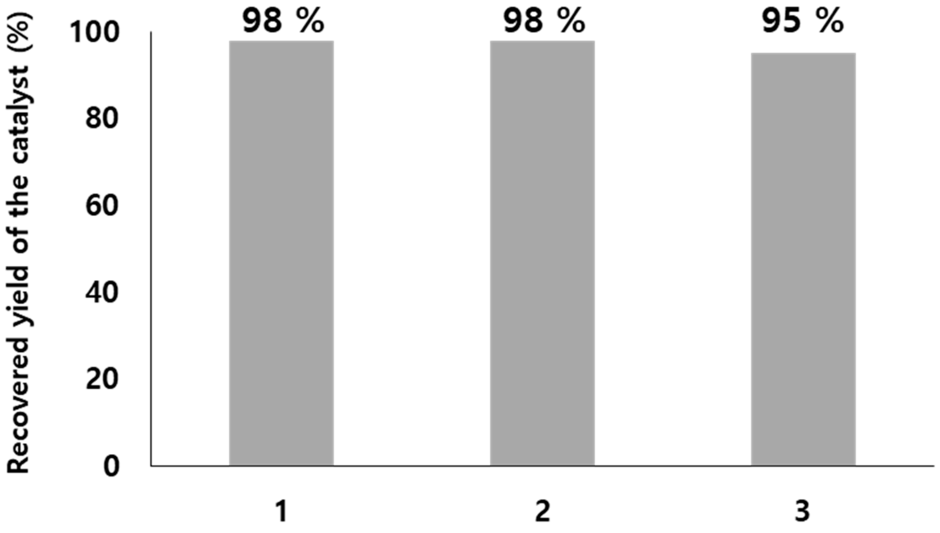

Figure S3. Recovered yield of the AEA-β-CD.

3. Binding constant of the AEA-β-CD

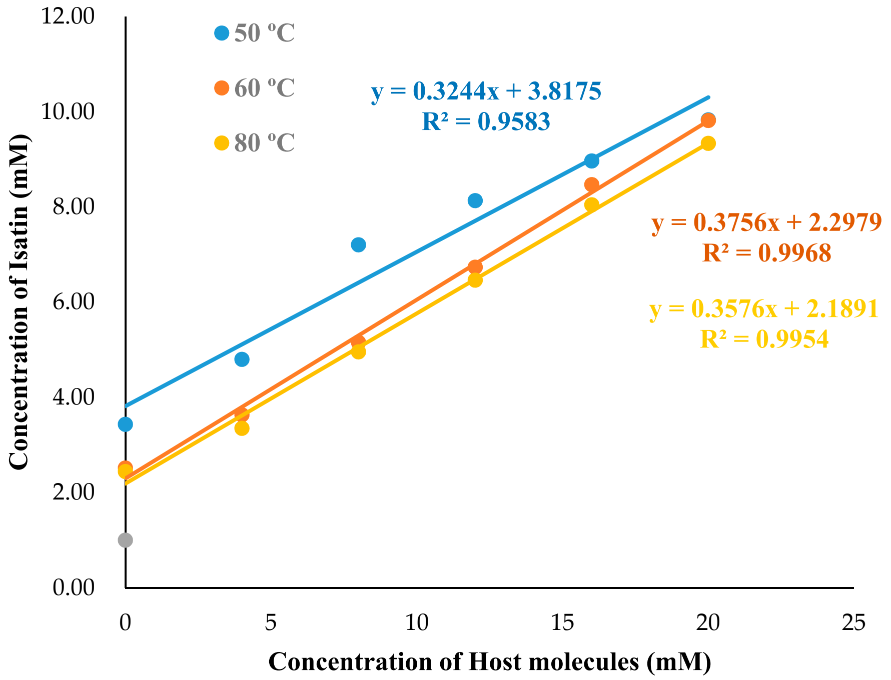

Figure S4. Phase solubility diagrams of isatin in aqueous solution with AEA-β-CD

	0	4	8	12	16	20
50 °C	3.4329	4.8002	7.2055	8.1330	8.9647	9.8312
60 °C	2.5155	3.6335	5.1565	6.7339	8.4684	9.8161
80 °C	2.4421	3.3486	4.9571	6.4625	8.0455	9.3367

Standard curve : y = 6.6227x + 0.0826

The binding constant, Kc, for the complex formation was calculated from the linear portion of the solubility diagram using the Higuchi and Connors equation.

$$K_c = \frac{\text{Slope}}{S_0(1-\text{Slope})}$$

Table S1. The binding constant Kc between isatine and AEA-β-CD

T(K)	S_0	slope	$K_c(M^{-1})$
353.15	2.4421	0.3576	227.9406
333.15	2.5155	0.3756	239.1361
323.15	3.4329	0.3244	139.8711

4. Spectral data of the Compounds

diethyl 2-propylquinoline-3,4-dicarboxylate (**4a**)

Yield 87 %; ^{1}H NMR (500 MHz, $CDCl_3$): δ 8.14 (1 H, d, *J* = 8.3), 8.08 (1 H, d, *J* = 8.5), 7.86 – 7.81 (1 H, m), 7.66 – 7.62 (1 H, m), 4.56 (2 H, q, *J* = 7.2), 4.49 (2 H, q, *J* = 7.2), 3.18 – 3.14 (2 H, m), 1.95 – 1.86 (2 H, m), 1.49 (6 H, dt, *J* = 9.5, 7.2), 1.09 (3 H, t, *J* = 7.4).13C NMR (125 MHz, $CDCl_3$) δ 167.38, 166.51, 159.53, 148.36, 138.88, 131.18, 129.41, 127.61, 125.51, 122.06, 110.57, 62.46, 62.18, 39.35, 23.20, 14.28, 14.25, 14.17. HRMS (ESI, m/z): calcd for C18H21NO4 (M+H+) 315.1471, found 315.1472.

diethyl 6-bromo-2-propylquinoline-3,4-dicarboxylate (**4b**)

Yield 89 %; ^{1}H NMR (500 MHz, $CDCl_3$): δ 8.29 (1 H, d, *J* = 2.1), 7.99 (1 H, d, *J* = 9.0), 7.88 (1 H, dd, *J* = 9.0, 2.1), 4.55 (2 H, q, *J* = 7.2), 4.48 (2 H, q, *J* = 7.2), 3.14 – 3.08 (2 H, m), 1.92 – 1.84 (2 H, m), 1.48 (6 H, dt, *J* = 10.5, 7.2), 1.07 (3 H, t, *J* = 7.4).13C NMR (125 MHz, $CDCl_3$) δ 167.09, 165.81, 159.85, 146.91, 137.24, 134.56, 131.04, 127.74, 125.78, 123.20, 121.84, 62.69, 62.31, 39.18, 22.90, 14.21, 14.19, 14.14. HRMS (ESI, m/z): calcd for C18H20BrNO4 (M+H+) 393.0576, found 393.0578.

diethyl 6-methoxy-2-propylquinoline-3,4-dicarboxylate (**4c**)

Yield 90 %; ^{1}H NMR (500 MHz, $CDCl_3$): δ 7.97 (1 H, d, *J* = 9.2), 7.42 (1 H, dd, *J* = 9.2, 2.8), 7.35 (1 H, d, *J* = 2.7), 4.49 (2 H, q, *J* = 7.2), 4.43 (2 H, q, *J* = 7.2), 3.92 (3 H, s), 3.08 – 3.01 (2 H, m), 1.86 – 1.78 (2 H, m), 1.43 (6 H, dt, *J* = 10.6, 7.2), 1.02 (3 H, t, *J* = 7.4).13C NMR (125 MHz, $CDCl_3$) δ 167.75, 166.67, 158.63, 156.64, 144.85, 136.74, 130.84, 125.24, 124.06, 123.24, 102.96, 62.30, 62.09, 55.68, 39.01, 23.28, 14.27, 14.25, 14.18. HRMS (ESI, m/z): calcd for C19H23NO5 (M+H+) 345.1576, found 345.1576.

diethyl 6-fluoro-2-propylquinoline-3,4-dicarboxylate (**4d**)

Yield 86 %; ^{1}H NMR (500 MHz, $CDCl_3$): δ 8.08 (1 H, dd, *J* = 9.3, 5.5), 7.74 (1 H, dd, *J* = 9.8, 2.8), 7.57 – 7.51 (1 H, m), 4.50 (2 H, q, *J* = 7.2), 4.44 (2 H, q, *J* = 7.2), 3.10 – 3.03 (2 H, m), 1.87 – 1.78 (2 H, m), 1.43 (6 H, dt, *J* = 10.0, 7.2), 1.02 (3 H, t, *J* = 7.4).13C NMR (125 MHz, $CDCl_3$) δ 167.34, 166.01, 162.14, 160.16, 158.68, 158.66, 145.59, 132.00, 131.93, 121.53, 121.32, 109.35, 109.15,

62.66, 62.29, 39.06, 23.02, 14.24, 14.22, 14.17. HRMS (ESI, m/z): calcd for C18H20FNO4 (M+H+) 333.3594, found 333.3595.

diethyl 6-nitro-2-propylquinoline-3,4-dicarboxylate (**4e**)

Yield 77 %; ^{1}H NMR (500 MHz, $CDCl_3$): δ 9.09 (1 H, d, *J* = 2.4), 8.53 (1 H, dd, *J* = 9.2, 2.5), 8.21 (1 H, d, *J = 2.5*), 4.57 (2 H, q, *J* = 7.2), 4.47 (2 H, q, *J* = 7.2), 3.14 – 3.07 (2 H, m), 1.93 – 1.83 (2 H, m), 1.48 (3 H, t, *J* = 8.2, 6.1), 1.44 (3 H, t, *J* = 7.2), 1.04 (3 H, t, *J* = 7.4).13C NMR (125 MHz, $CDCl_3$) δ 166.66, 165.19, 163.58, 150.05, 146.24, 139.34, 131.32, 124.40, 122.90, 121.25, 63.21, 62.64, 39.40, 22.69, 14.24, 14.23, 14.16. HRMS (ESI, m/z): calcd for C18H20N2O6 (M+H+) 360.3660, found 360.3658.

diethyl 2-methylquinoline-3,4-dicarboxylate (**4f**)

Yield 92 %; ^{1}H NMR (500 MHz, $CDCl_3$): δ 8.06 (1 H, d, *J* = 8.4), 8.01 (1 H, d, *J* = 8.5), 7.81 – 7.75 (1 H, m), 7.61 – 7.56 (1 H, m), 4.51 (2 H, q, *J* = 7.2), 4.44 (2 H, q, *J* = 7.2), 2.86 (3 H, s), 1.43 (6 H, dt, *J* = 9.3, 7.2).13C NMR (125 MHz, $CDCl_3$) δ 167.14, 166.48, 156.09, 148.33, 139.20, 131.39, 129.23, 127.65, 125.58, 124.29, 122.14, 77.16, 62.48, 62.21, 24.75, 14.25, 14.21. HRMS (ESI, m/z): calcd for C16H17NO4 (M+H+) 287.1158, found 287.1159.

diethyl 6-chloro-2-methylquinoline-3,4-dicarboxylate (**4g**)

Yield 91 %; ^{1}H NMR (500 MHz, $CDCl_3$): δ 8.06 (1 H, d, *J* = 2.8), 8.00 (1 H, d, *J* = 9.0), 7.71 (1 H, dd, *J* = 9.0, 2.3), 4.51 (2 H, q, *J* = 7.2), 4.44 (2 H, q, *J* = 7.2), 2.83 (3 H, s), 1.43 (6 H, dt, *J* = 9.9, 7.2).13C NMR (125 MHz, $CDCl_3$) δ 166.97, 165.84, 156.31, 146.72, 137.64, 133.73, 132.27, 130.81, 124.56, 122.86, 62.75, 62.38, 29.84, 24.56, 14.21.HRMS (ESI, m/z): calcd for C16H16ClNO4 (M+H+) 321.0768, found 321.0770.

diethyl 6-methoxy-2-methylquinoline-3,4-dicarboxylate (**4h**)

Yield 92 %; ^{1}H NMR (500 MHz, $CDCl_3$): δ 7.95 (1 H, d, *J* = 9.2), 7.43 (1 H, dd, *J* = 9.2, 2.8), 7.34 (1 H, d, *J* = 2.7), 4.50 (2 H, q, *J* = 7.2), 4.43 (2 H, q, *J* = 7.2), 3.92 (3 H, s), 2.80 (3 H, s), 1.43 (6 H, dt, *J* = 10.3, 7.2).13C NMR (125 MHz, $CDCl_3$) δ 167.52, 166.62, 158.63, 153.07, 144.76, 137.02,

130.64, 125.03, 124.21, 123.31, 103.05, 62.31, 62.13, 55.68, 24.23, 14.24, 14.21.HRMS (ESI, m/z): calcd for C17H19NO5 (M+H+) 317.1263, found 317.1265.

diethyl 6-fluoro-2-methylquinoline-3,4-dicarboxylate (**4i**)

Yield 90 %; ^{1}H NMR (500 MHz, $CDCl_3$): δ 8.06 (1 H, dd, *J* = 9.2, 5.4), 7.73 (1 H, dd, *J* = 9.7, 2.8), 7.58 – 7.52 (1 H, m), 4.53 – 4.48 (2 H, m), 4.44 (2 H, q, *J* = 7.2), 2.82 (3 H, s), 1.43 (6 H, dt, *J* = 9.3, 7.2).13C NMR (125 MHz, $CDCl_3$) δ 167.11, 165.92, 162.11, 160.13, 155.18, 155.16, 145.49, 131.78, 131.71, 121.68, 121.48, 109.42, 109.23, 62.66, 62.32, 24.34, 14.19, 14.17..HRMS (ESI, m/z): calcd for C16H16FNO4 (M+H+) 305.1063, found 305.1061.

diethyl 6-bromo-2-methylquinoline-3,4-dicarboxylate (**4j**)

Yield 90 %; ^{1}H NMR (500 MHz, $CDCl_3$): δ 8.22 (1 H, d, *J* 2.1), 7.92 (1 H, d, *J* 9.0), 7.84 (1 H, dd, *J* 9.0, 2.1), 4.53 – 4.48 (2 H, m), 4.44 (2 H, q, *J* 7.2), 2.82 (3 H, s), 1.43 (6 H, dt, *J* 9.9, 7.2).13C NMR (125 MHz, $CDCl_3$) δ 166.92, 165.80, 156.45, 146.90, 137.55, 134.81, 130.88, 127.84, 125.59, 123.32, 121.91, 62.75, 62.39, 24.60, 14.20. HRMS (ESI, m/z): calcd for C16H16BrNO4 (M+H+) 365.0263, found 365.0261.

ethyl 3-acetyl-6-fluoro-2-methylquinoline-4-carboxylate (**4k**)

Yield 92 %; ^{1}H NMR (500 MHz, $CDCl_3$): δ 8.45 (1 H, dd, *J* = 8.8, 2.9), 8.15 (1 H, dt, *J* = 10.8, 5.4), 7.64 – 7.57 (1 H, m), 3.53 (1 H, dq, *J* = 8.8, 7.1), 3.11 (1 H, dq, *J* = 8.9, 7.0), 2.85 (3 H, s), 1.98 (3 H, s), 1.22 (3 H, t, *J* = 7.0).13C NMR (125 MHz, $CDCl_3$) δ 166.92, 163.01, 161.00, 152.94, 152.92, 146.54, 141.47, 131.73, 131.66, 121.64, 121.44, 107.93, 107.74, 60.39, 24.55, 21.78, 15.18. HRMS (ESI, m/z): calcd for C15H14FNO3 (M+H+) 275.0958, found 275.0960.

ethyl 3-acetyl-2-methylquinoline-4-carboxylate (**4l**)

Yield 92 %; ^{1}H NMR (500 MHz, $CDCl_3$): δ 8.84 (1 H, d, *J* = 8.3), 8.16 (1 H, d, *J* = 8.5), 7.88 – 7.82 (1 H, m), 7.74 – 7.68 (1 H, m), 3.52 (1 H, dq, *J* = 8.9, 7.1), 3.10 (1 H, dq, *J* = 8.9, 7.0), 2.86 (3 H, s), 1.98 (3 H, s), 1.21 (3 H, t, *J* = 7.0).13C NMR (125 MHz, $CDCl_3$) δ 167.20, 153.71, 149.36, 140.70, 131.24, 130.30, 129.13, 128.83, 123.83, 121.64, 108.58, 60.28, 24.67, 21.95, 15.18.HRMS (ESI, m/z): calcd for C15H15NO3 (M+H+) 257.1052, found 257.1050.

ethyl 3-acetyl-6-bromo-2-methylquinoline-4-carboxylate (**4m**)

Yield 90 %; ^{1}H NMR (500 MHz, $CDCl_3$): δ 9.01 (1 H, d, *J* = 2.2), 8.01 (1 H, d, *J* = 9.0), 7.91 (1 H, dd, *J* = 9.0, 2.2), 3.52 (1 H, dq, *J* = 8.8, 7.1), 3.10 (1 H, dq, *J* = 8.9, 7.0), 2.83 (3 H, s), 1.97 (3 H, s), 1.22 (3 H, t, *J* = 7.0).13C NMR (125 MHz, $CDCl_3$) δ 166.75, 154.21, 147.94, 141.49, 134.92, 130.69, 129.43, 126.22, 123.54, 122.63, 108.67, 60.42, 24.55, 21.99, 15.19.HRMS (ESI, m/z): calcd for C15H14BrNO3 (M+H+) 335.0157, found 335.0156.

ethyl 3-acetyl-6-chloro-2-methylquinoline-4-carboxylate (**4n**)

Yield 89 %; ^{1}H NMR (500 MHz, $CDCl_3$): δ 8.82 (1 H, d, *J* = 2.3), 8.08 (1 H, d, *J* = 9.0), 7.80 – 7.74 (1 H, m), 3.56 – 3.49 (1 H, m), 3.14 – 3.08 (1 H, m), 2.85 (3 H, s), 1.98 (3 H, s), 1.22 (3 H, t, *J* = 7.0).13C NMR (125 MHz, $CDCl_3$) δ 166.75, 154.01, 147.70, 141.51, 135.21, 132.29, 130.60, 129.55, 122.87, 122.19, 108.65, 60.40, 24.52, 21.92, 15.18. HRMS (ESI, m/z): calcd for C15H14ClNO3 (M+H+) 291.0662, found 291.0664.

isopropyl 3-acetyl-2-methylquinoline-4-carboxylate (**4o**)

Yield 91 %; ^{1}H NMR (500 MHz, $CDCl_3$): δ 8.86 (1 H, dd, *J* = 8.3, 0.9), 8.16 (1 H, d, *J* = 8.5), 7.87 – 7.83 (1 H, m), 7.74 – 7.70 (1 H, m), 3.68 – 3.59 (1 H, m), 2.89 (3 H, s), 1.97 (3 H, s), 1.25 (3 H, d, *J* = 6.1), 1.06 (3 H, d, *J* = 6.2).13C NMR (125 MHz, $CDCl_3$) δ 167.35, 153.91, 149.31, 141.08, 131.22, 129.13, 128.84, 123.90, 121.58, 108.70, 68.58, 24.92, 24.41, 23.44, 22.23. HRMS (ESI, m/z): calcd for C16H17NO3 (M+H+) 271.3160, found 271.3162.

isopropyl 3-acetyl-6-fluoro-2-methylquinoline-4-carboxylate (**4p**)

Yield 90 %; ^{1}H NMR (500 MHz, $CDCl_3$): δ 8.47 (2 H, dd, *J* = 8.9, 2.9), 8.16 (2 H, dd, *J* = 9.3, 5.2), 7.63 – 7.57 (2 H, m), 3.65 (2 H, hept, *J* = 6.1), 2.87 (6 H, s), 1.97 (6 H, s), 1.25 (7 H, d, *J* = 6.1), 1.07 (6 H, d, *J* = 6.2).13C NMR (125 MHz, $CDCl_3$) δ 167.05, 163.03, 161.03, 153.11, 153.09, 146.47, 141.83, 131.71, 131.64, 121.60, 121.40, 107.97, 107.78, 68.72, 24.75, 24.37, 23.45, 22.04. HRMS (ESI, m/z): calcd for C16H17NO3 (M+H+) 271.3160, found 271.3162.

isopropyl 3-acetyl-6-methoxy-2-methylquinoline-4-carboxylate (**4q**)

Yield 87 %; ^{1}H NMR (500 MHz, $CDCl_3$): δ 8.13 (1 H, d, *J* = 2.8), 8.03 (1 H, d, *J* = 9.3), 7.47 (1 H, dd, *J* = 9.3, 2.9), 4.00 (3 H, s), 3.64 – 3.58 (1 H, m), 2.83 (3 H, s), 1.96 (3 H, s), 1.24 (3 H, d, *J* = 6.1), 1.06 (3 H, d, *J* = 6.2).13C NMR (125 MHz, $CDCl_3$) δ 167.76, 159.86, 150.56, 145.64, 140.98, 130.53, 128.80, 124.32, 123.12, 108.71, 101.19, 68.53, 56.06, 24.99, 24.41, 23.42, 21.78.HRMS (ESI, m/z): calcd for C17H19NO4 (M+H+) 301.3420, found 301.3420.

isopropyl 3-acetyl-8-fluoro-2-methylquinoline-4-carboxylate (**4r**)

Yield 69 %; ^{1}H NMR (500 MHz, $CDCl_3$): δ 8.64 (1 H, d, *J* = 8.3), 7.67 (1 H, td, *J* = 8.1, 4.9), 7.54 (1 H, ddd, *J* = 10.6, 7.8, 1.2), 3.65 (1 H, dt, *J* = 12.3, 6.1), 2.94 (3 H, s), 1.98 (3 H, s), 1.26 (3 H, d, *J* = 6.1), 1.07 (3 H, d, *J* = 6.2).13C NMR (125 MHz, $CDCl_3$) δ 166.90, 158.65, 156.59, 154.54, 142.12, 139.39, 139.30, 130.19, 130.17, 129.02, 128.95, 123.10, 119.75, 119.71, 115.54, 115.39, 108.68, 68.74, 24.72, 24.36, 23.42, 22.39.HRMS (ESI, m/z): calcd for C17H18ClNO3 (M+H+) 319.7850, found 319.7848.

butyl 3-acetyl-2-methylquinoline-4-carboxylate (**4s**)

Yield 84 %; ^{1}H NMR (500 MHz, $CDCl_3$): δ 8.84 (1 H, d, *J* = 8.3), 8.16 (1 H, d, *J* = 8.5), 7.88 – 7.82 (1 H, m), 7.74 – 7.70 (1 H, m), 3.45 (1 H, dt, *J* = 8.8, 6.5), 3.02 (1 H, dt, *J* = 8.8, 6.5), 2.86 (3 H, s), 1.98 (3 H, s), 1.60 – 1.52 (2 H, m), 1.41 – 1.29 (2 H, m), 0.88 (3 H, t, *J* = 7.4).13C NMR (125 MHz, $CDCl_3$) δ 167.22, 153.70, 149.35, 140.75, 131.21, 130.28, 129.11, 128.80, 123.84, 121.64, 108.58, 64.32, 31.60, 24.59, 21.96, 19.27, 13.86.HRMS (ESI, m/z): calcd for C16H17NO3 (M+H+) 271.3160, found 271.3162.

butyl 3-acetyl-6-bromo-2-methylquinoline-4-carboxylate (**4t**)

Yield 86 %; ^{1}H NMR (500 MHz, $CDCl_3$): δ 9.00 (1 H, d, *J* = 2.2), 8.01 (1 H, d, *J* = 9.0), 7.91 (1 H, dd, *J* = 9.0, 2.2), 3.45 (1 H, dt, *J* = 8.8, 6.5), 3.02 (1 H, dt, *J* = 8.8, 6.5), 2.83 (3 H, s), 1.97 (3 H, s), 1.59 – 1.53 (2 H, m), 1.40 – 1.31 (2 H, m), 0.88 (3 H, t, *J* = 7.4).13C NMR (125 MHz, $CDCl_3$) δ 166.76, 154.20, 147.90, 141.52, 134.87, 130.66, 129.40, 126.20, 123.49, 122.61, 108.66, 64.45, 31.58, 24.45, 21.98, 19.27, 13.86.HRMS (ESI, m/z): calcd for C17H18BrNO3 (M+H+) 364.2390, found 364.2392.

butyl 3-acetyl-6-fluoro-2-methylquinoline-4-carboxylate (**4u**)

Yield 83 %; ^{1}H NMR (500 MHz, $CDCl_3$): δ 8.44 (1 H, dd, *J* = 8.8, 2.9), 8.16 (1 H, dd, *J* = 9.3, 5.2), 7.60 (1 H, ddd, *J* = 9.2, 8.3, 2.9), 3.46 (1 H, dt, *J* = 8.8, 6.5), 3.04 (1 H, dt, *J* = 8.8, 6.5), 2.85 (3 H, s), 1.98 (3 H, s), 1.60 – 1.53 (2 H, m), 1.41 – 1.32 (2 H, m), 0.89 (3 H, t, *J* = 7.4).13C NMR (125 MHz, $CDCl_3$) δ 166.90, 162.95, 160.94, 152.91, 152.89, 146.49, 141.48, 131.69, 131.62, 130.00, 129.96, 122.52, 122.42, 121.56, 121.35, 108.64, 107.88, 107.69, 64.39, 31.56, 24.43, 21.76, 19.25, 13.83.HRMS (ESI, m/z): calcd for C17H18FNO3 (M+H+) 303.3334, found 303.3333.

butyl 3-acetyl-6-chloro-2-methylquinoline-4-carboxylate (**4v**)

Yield 85 %; ^{1}H NMR (500 MHz, $CDCl_3$): δ 8.82 (1 H, d, *J* = 2.3), 8.08 (1 H, d, *J* = 9.0), 7.77 (1 H, dd, *J* = 9.0, 2.4), 3.45 (1 H, dt, *J* = 8.8, 6.5), 3.03 (1 H, dt, *J* = 8.8, 6.5), 2.84 (3 H, s), 1.97 (3 H, s), 1.60 – 1.53 (2 H, m), 1.40 – 1.31 (2 H, m), 0.89 (3 H, t, *J* = 7.4).13C NMR (125 MHz, $CDCl_3$) δ 166.79, 154.01, 147.70, 141.56, 135.20, 132.28, 130.60, 129.54, 122.89, 122.21, 108.67, 64.44, 31.58, 24.45, 21.93, 19.27, 13.86. HRMS (ESI, m/z): calcd for C17H18ClNO3 (M+H+) 319.7850, found 319.7848.

butyl 3-acetyl-6-methoxy-2-methylquinoline-4-carboxylate (**4w**)

Yield 85 %; ^{1}H NMR (500 MHz, $CDCl_3$): δ 8.11 (1 H, d, *J* 2.8), 8.03 (1 H, d, *J* 9.3), 7.46 (1 H, dd, *J* 9.3, 2.9), 4.00 (3 H, s), 3.43 (1 H, dt, *J* 8.8, 6.5), 3.01 (1 H, dt, *J* 8.8, 6.5), 2.81 (3 H, s), 1.97 (3 H, s), 1.59 – 1.52 (2 H, m), 1.41 – 1.31 (2 H, m), 0.88 (3 H, t, *J* 7.4).13C NMR (125 MHz, $CDCl_3$) δ 167.64, 159.80, 150.36, 145.66, 140.65, 130.50, 128.76, 124.29, 123.18, 108.57, 101.14, 77.16, 64.25, 56.01, 31.60, 24.63, 21.48, 19.26, 13.85. HRMS (ESI, m/z): calcd for C18H21NO4 (M+H+) 315.3690, found 315.3691.

5. ¹H NMR and ¹³C NMR Spectra of all compounds

¹H and ¹³C NMR of compound **4a**

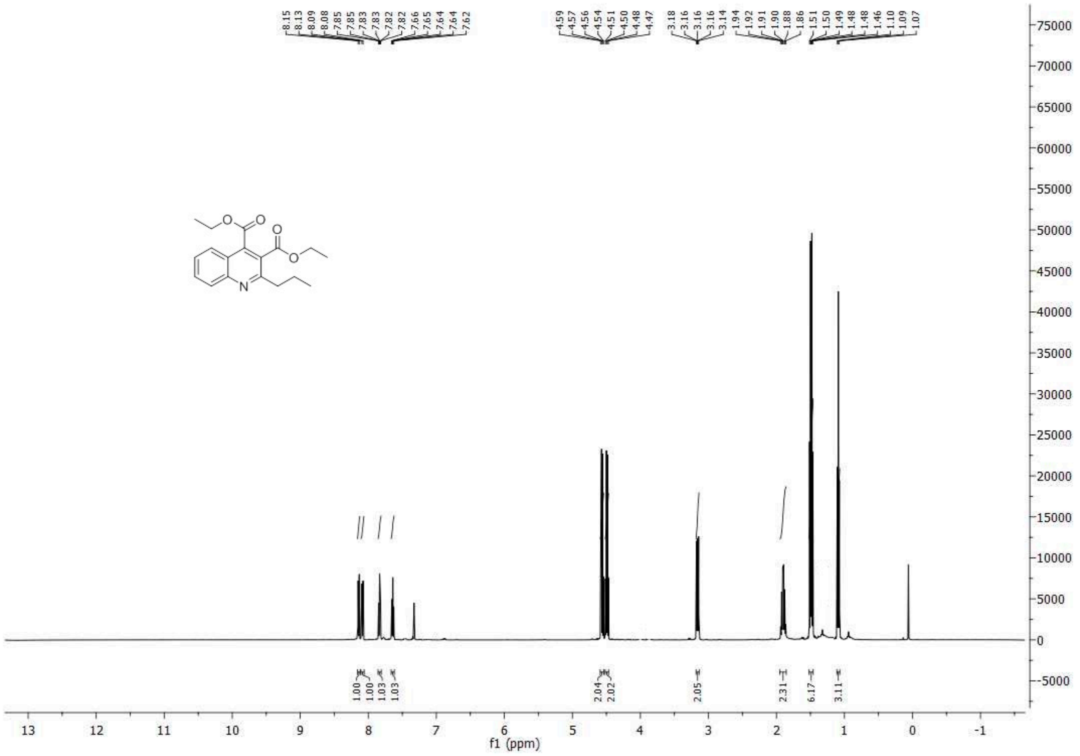

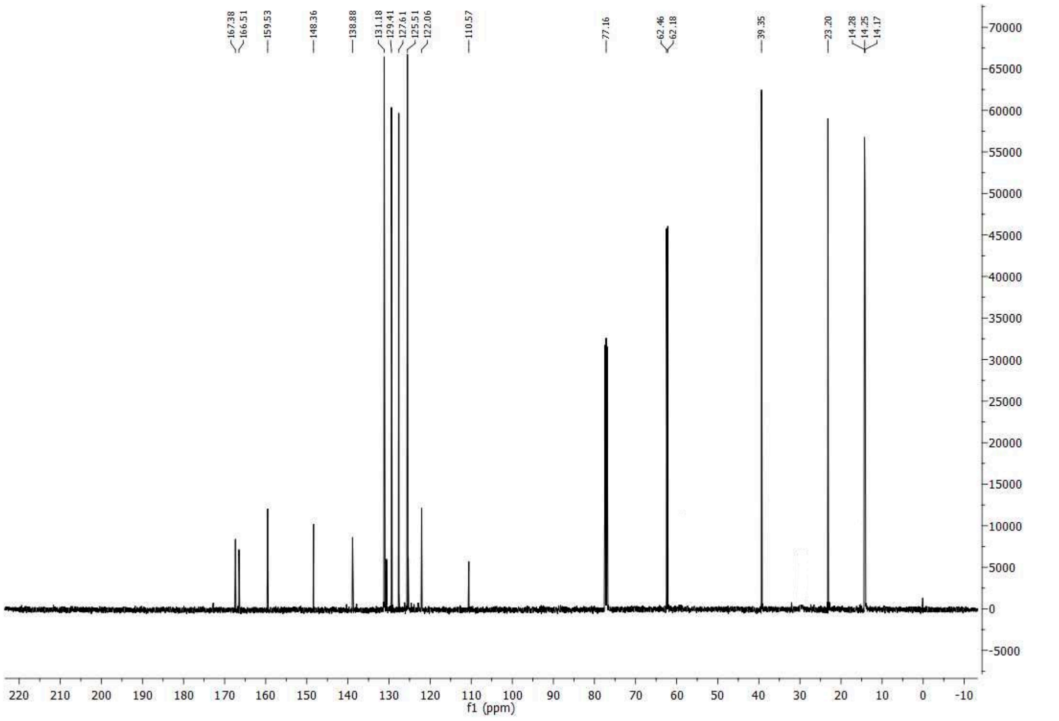

.

^{1}H and ^{13}C NMR of compound **4b**

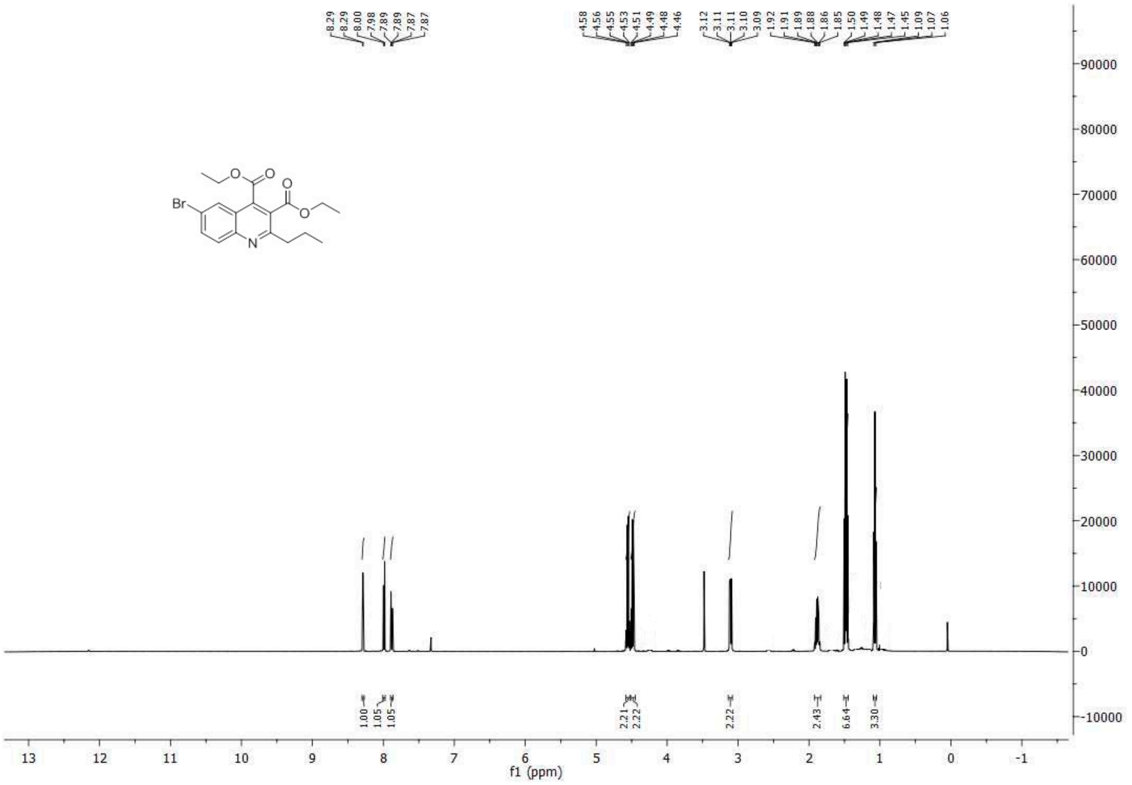

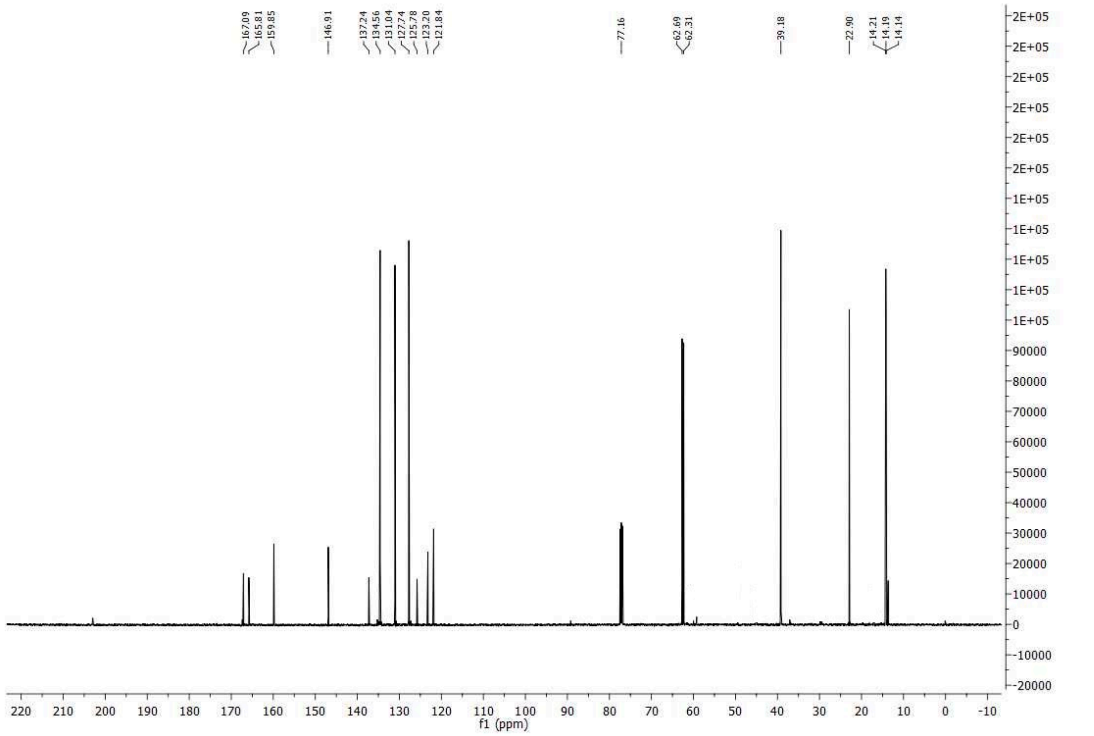

^{1}H and ^{13}C NMR of compound **4c**

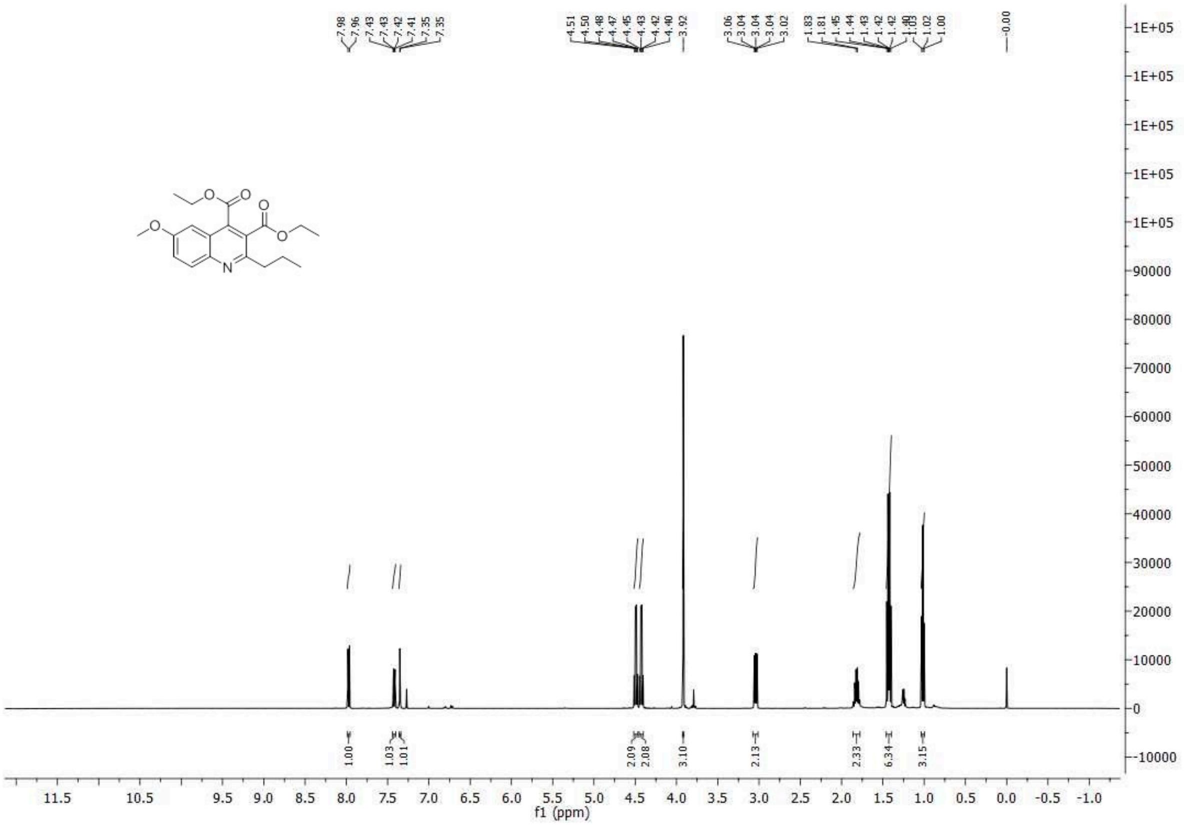

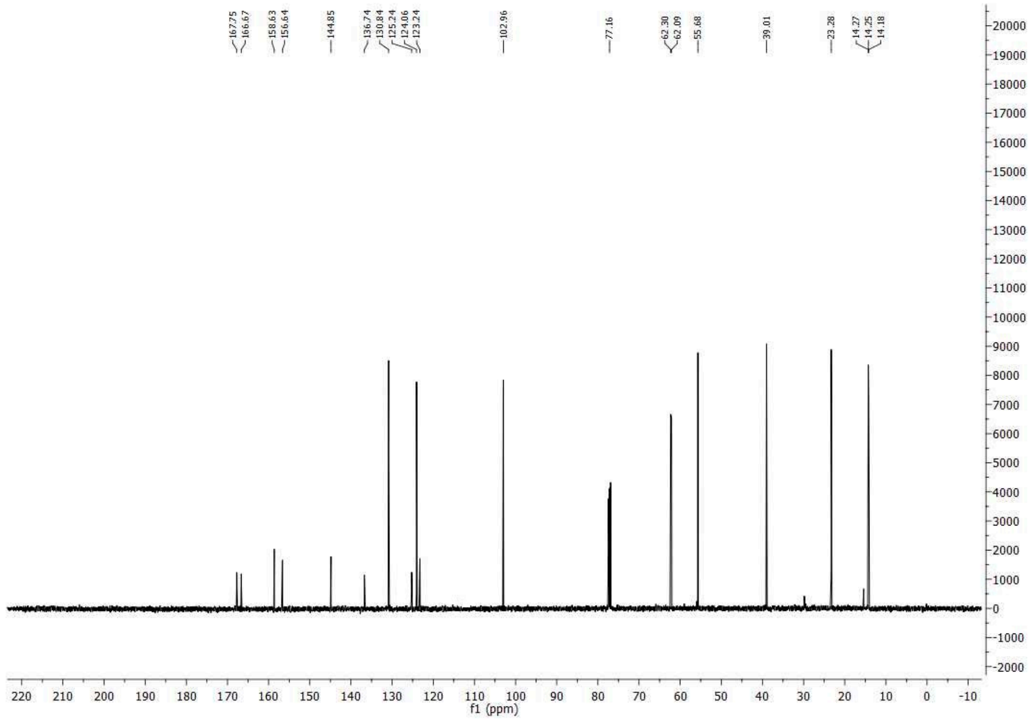

^{1}H and ^{13}C NMR of compound **4d**

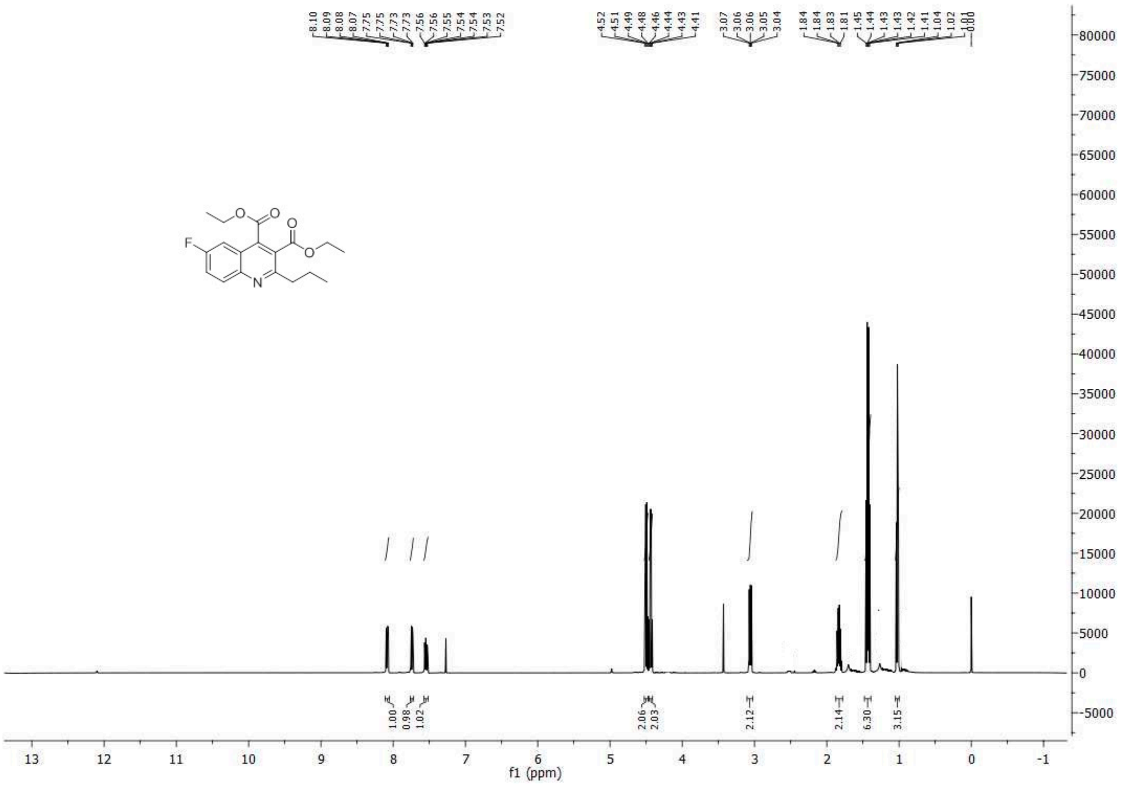

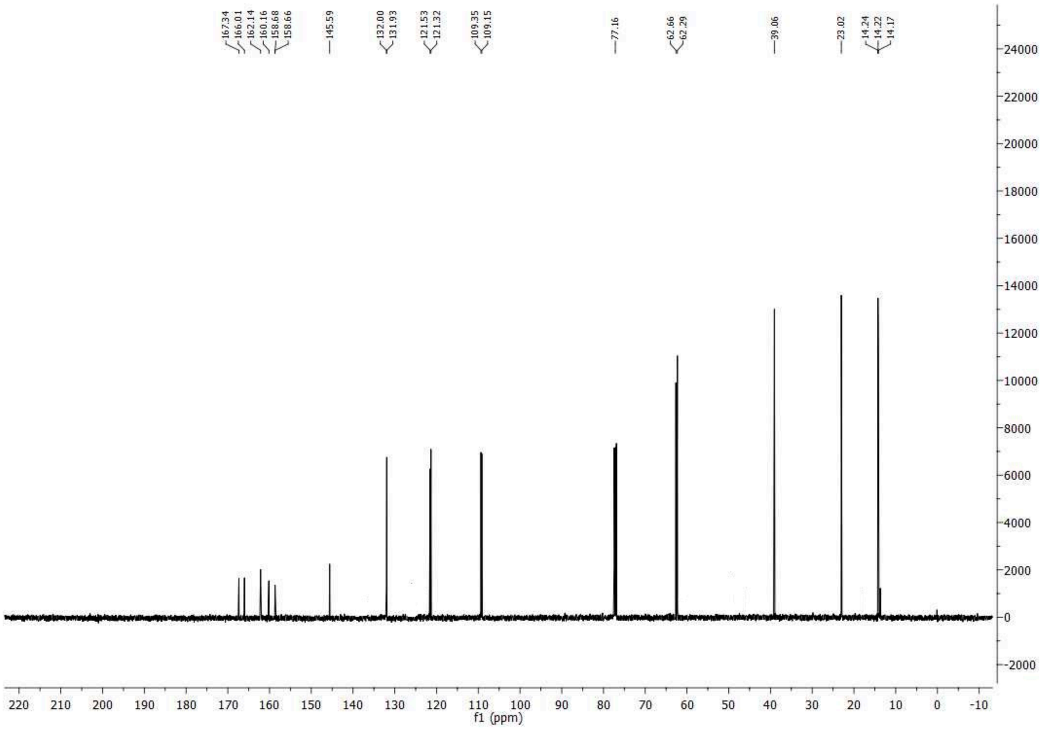

^{1}H and ^{13}C NMR of compound **4e**

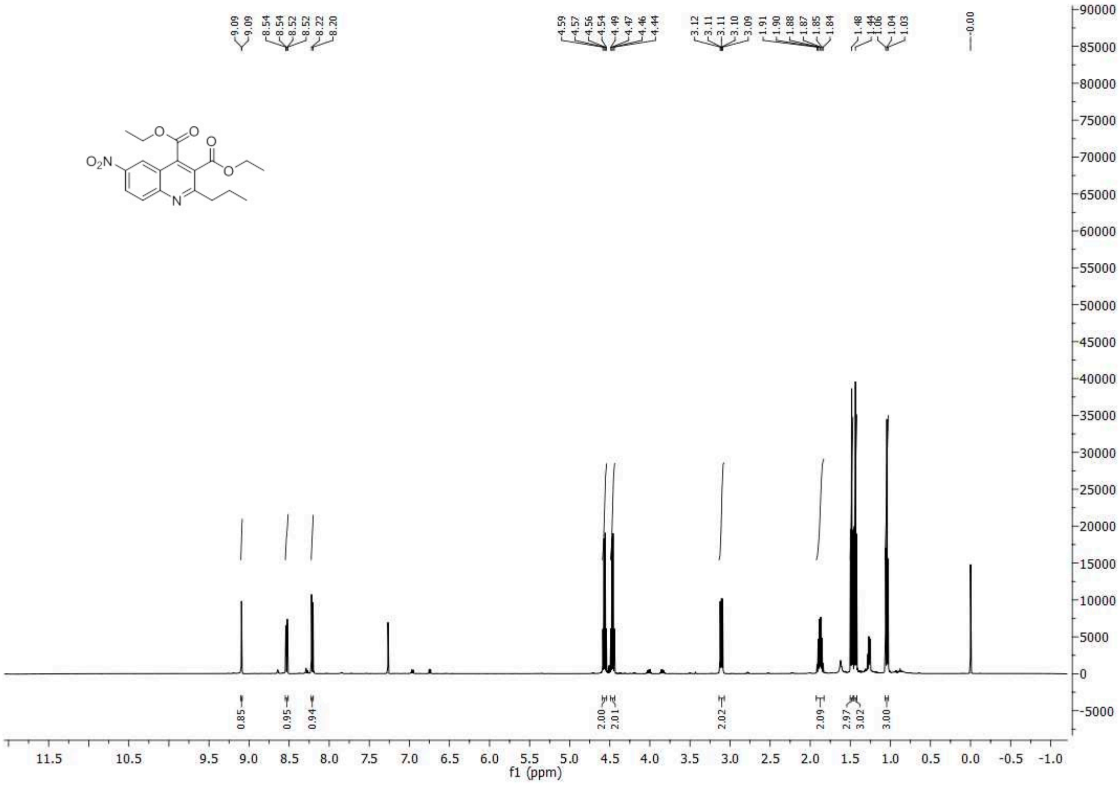

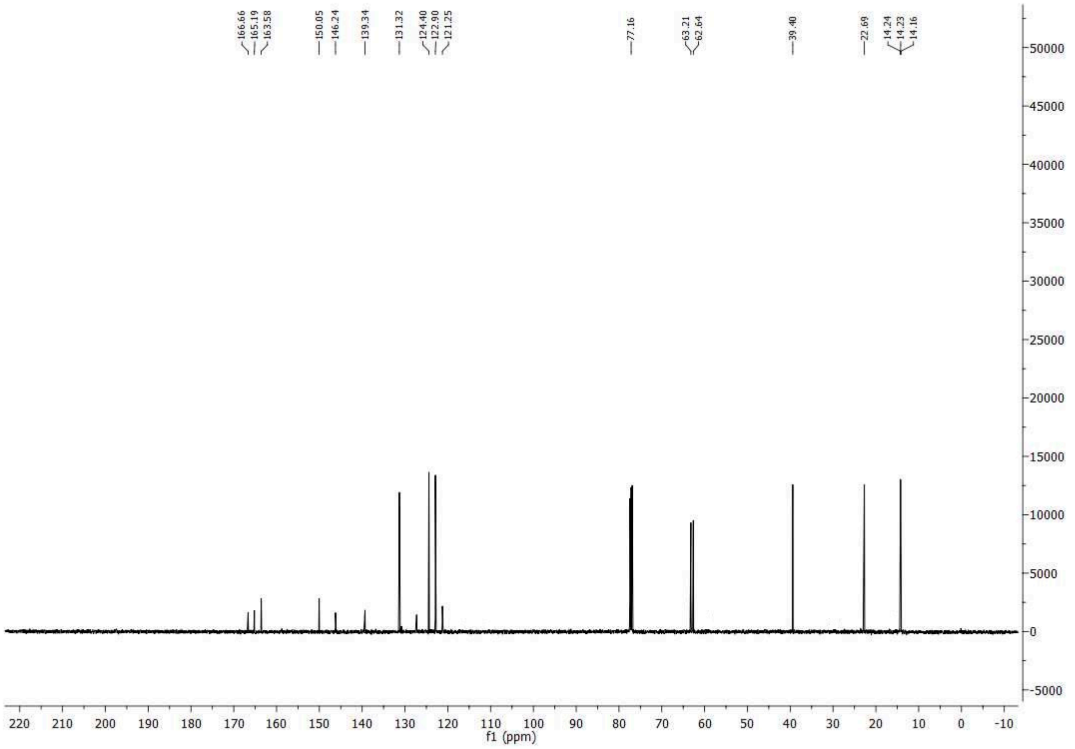

^{1}H and ^{13}C NMR of compound **4f**

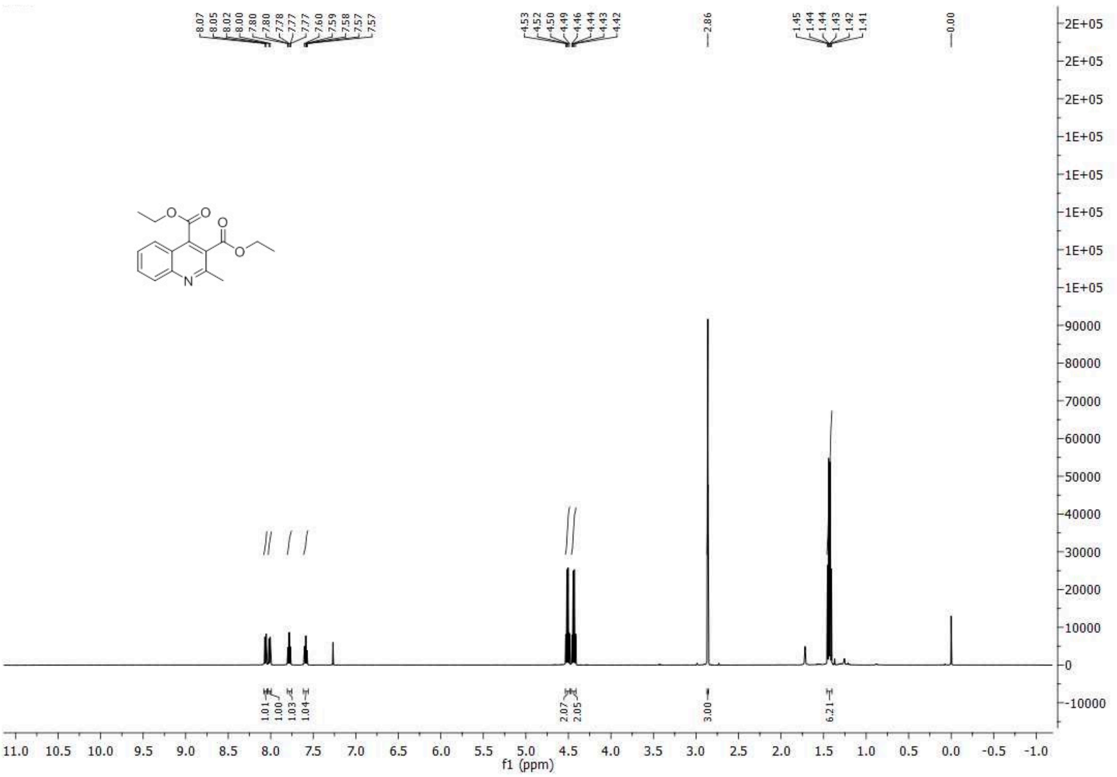

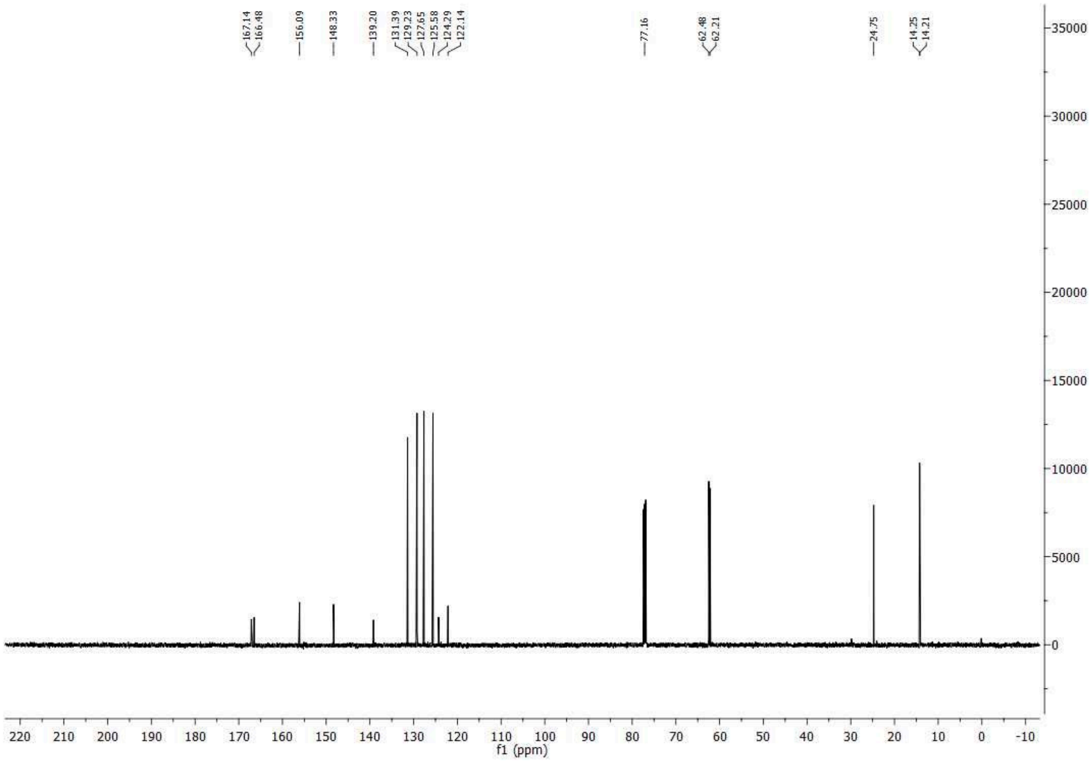

^{1}H and ^{13}C NMR of compound **4g**

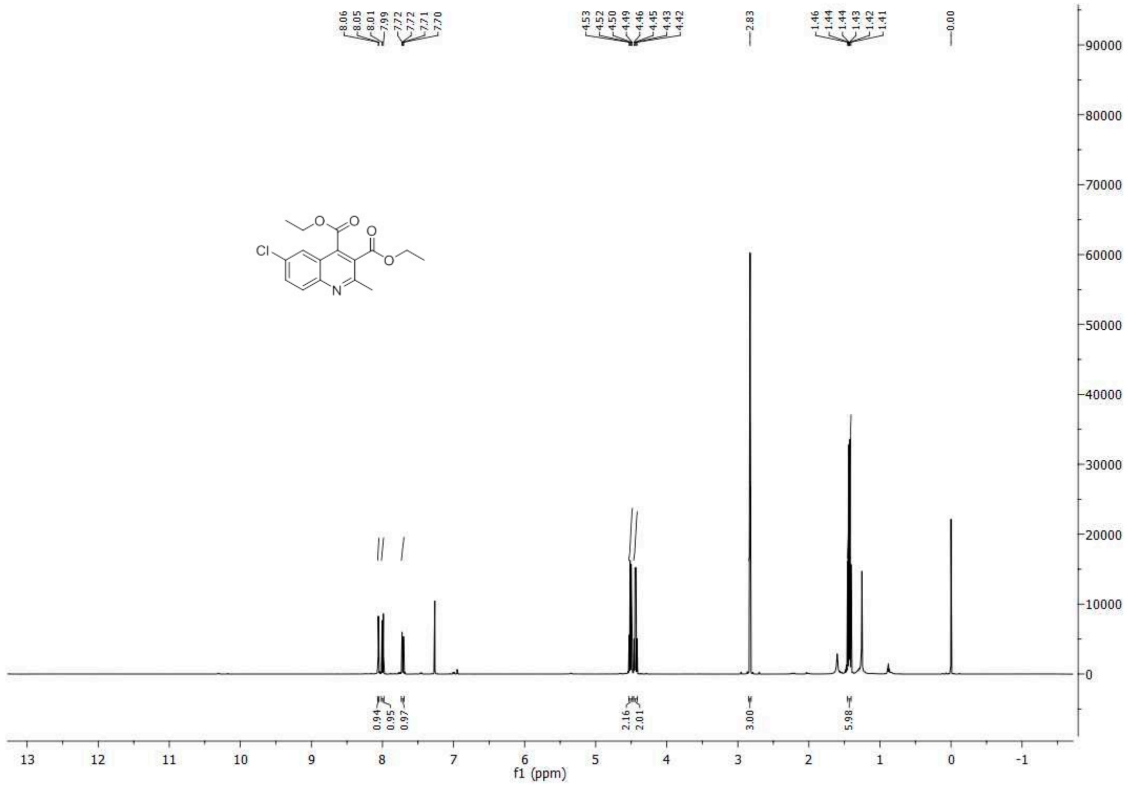

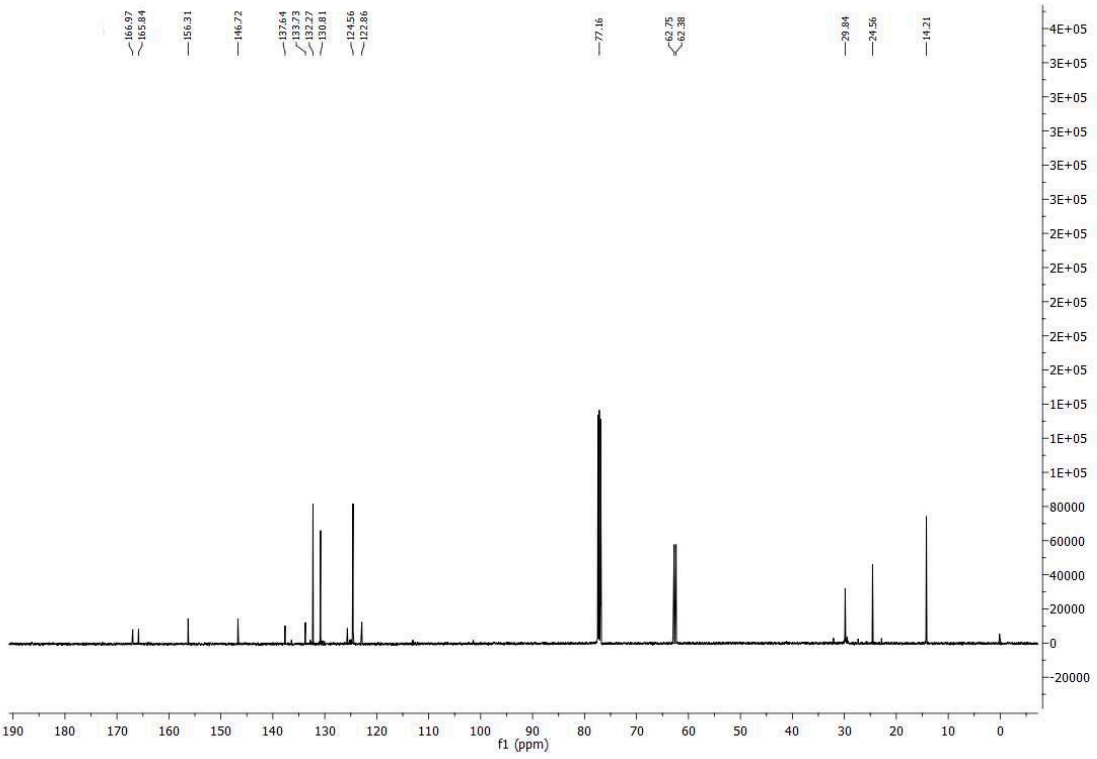

^{1}H and ^{13}C NMR of compound **4h**

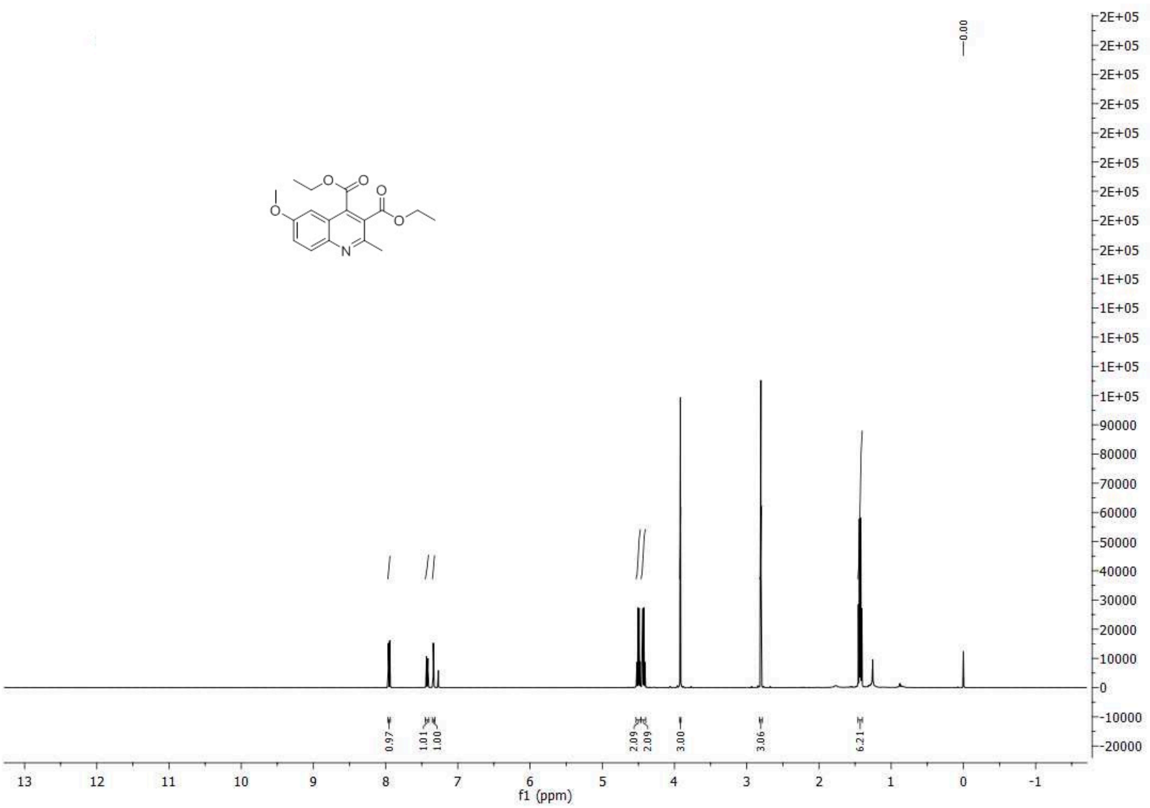

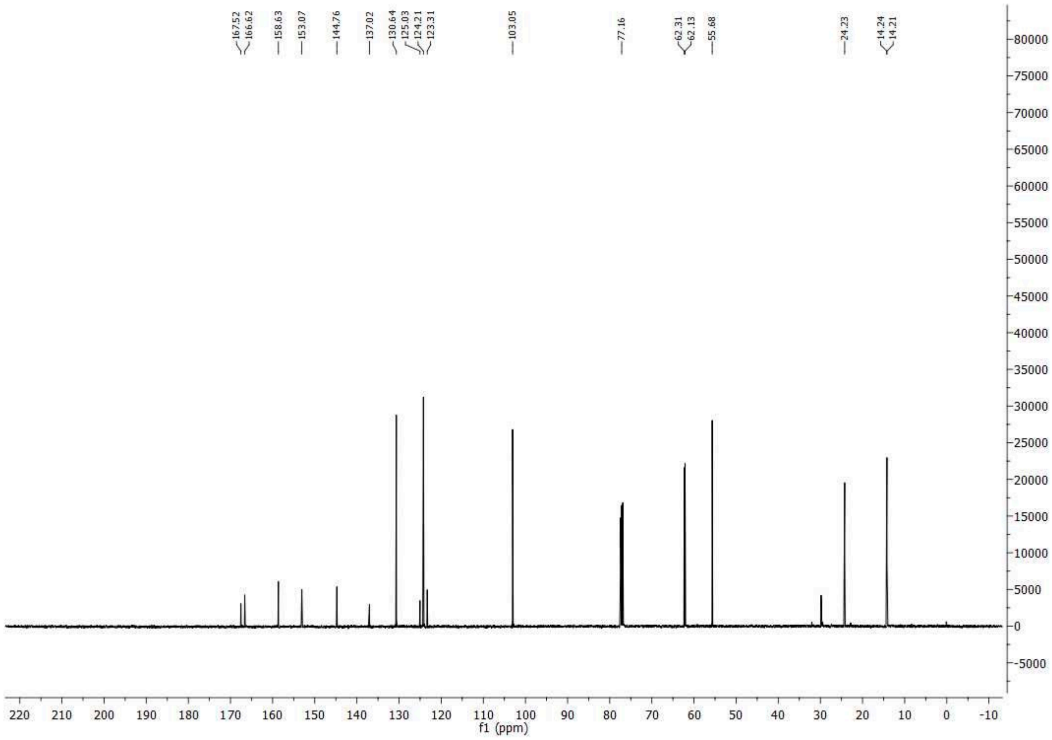

^{1}H and ^{13}C NMR of compound **4i**

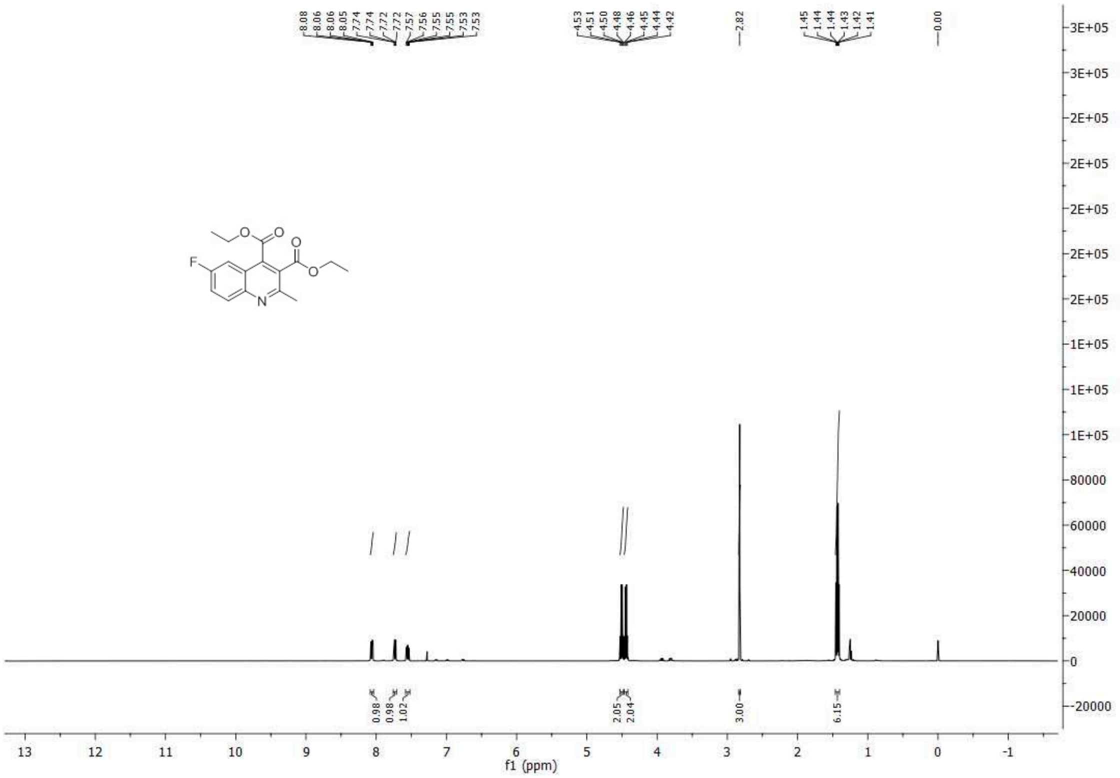

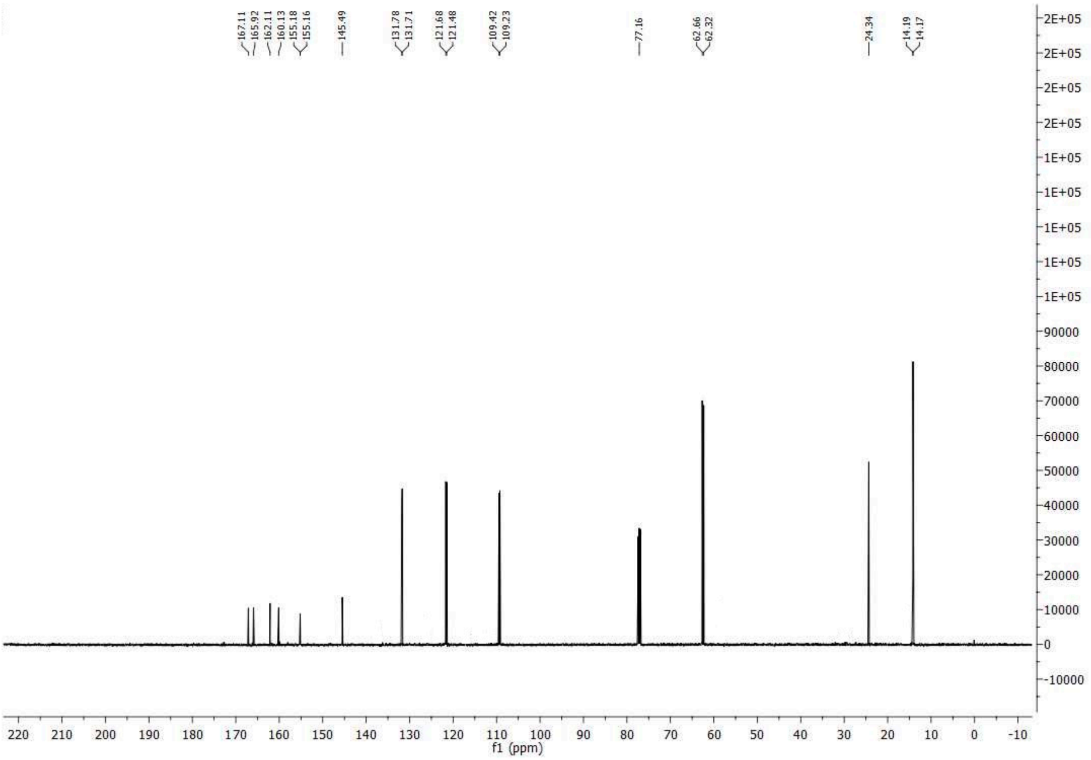

^{1}H and ^{13}C NMR of compound **4j**

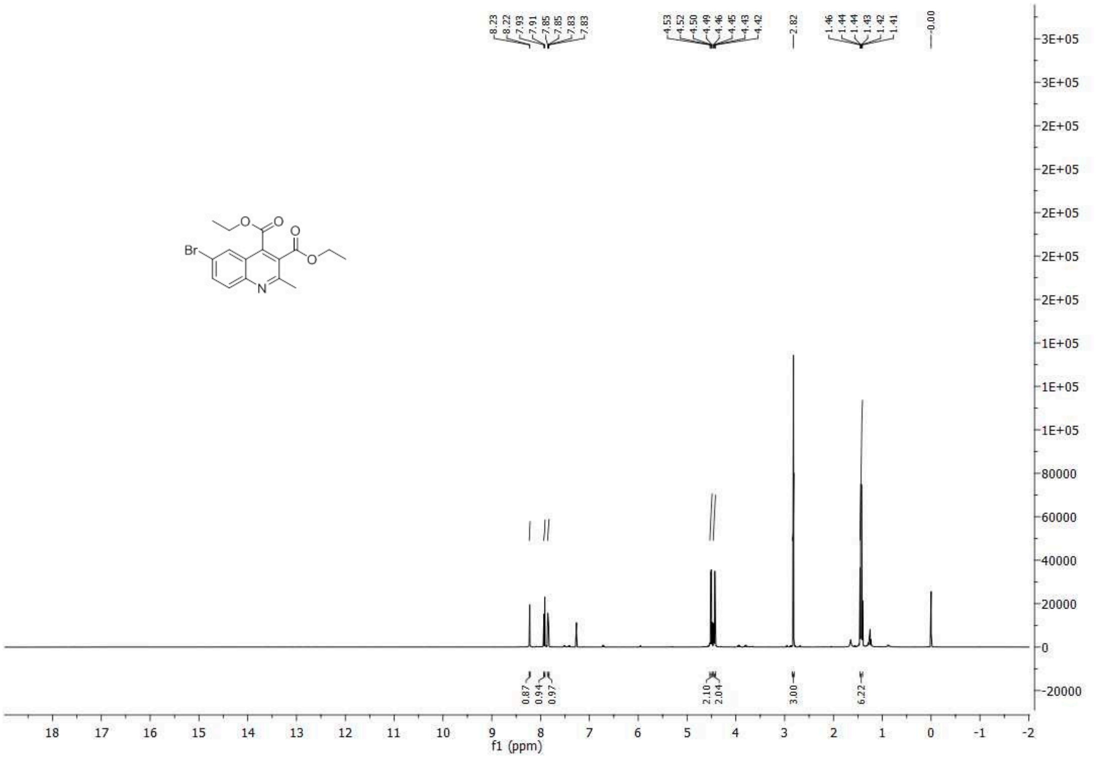

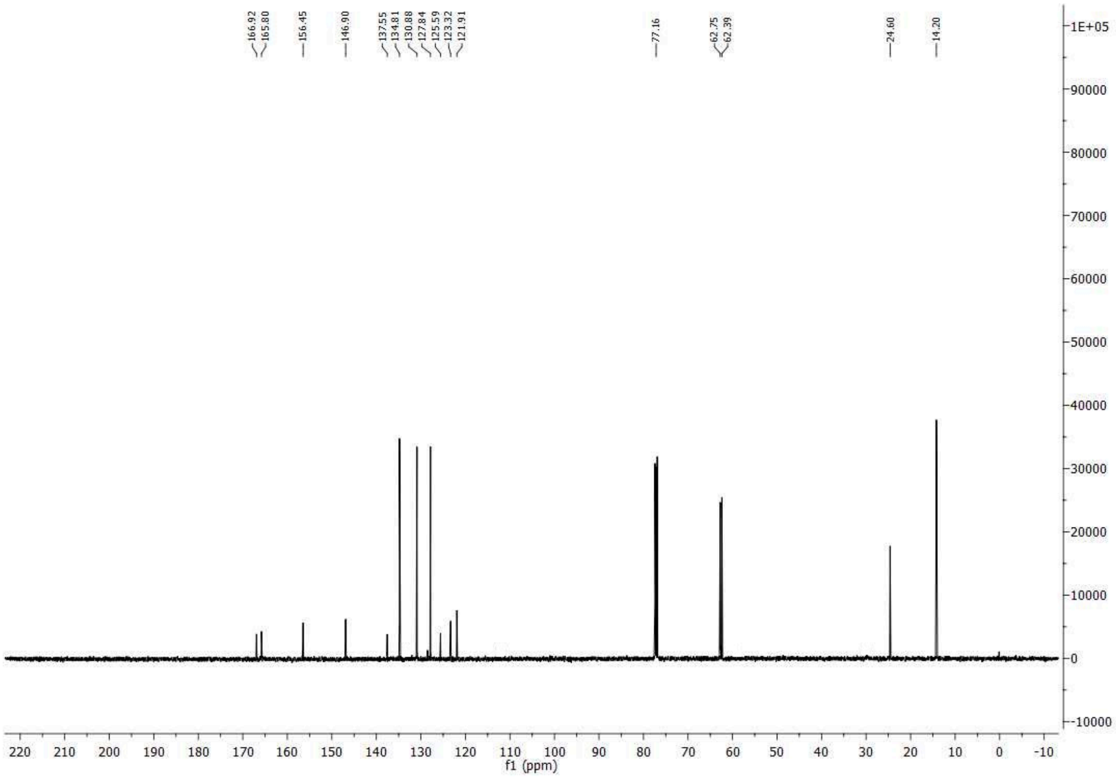

^{1}H and ^{13}C NMR of compound **4k**

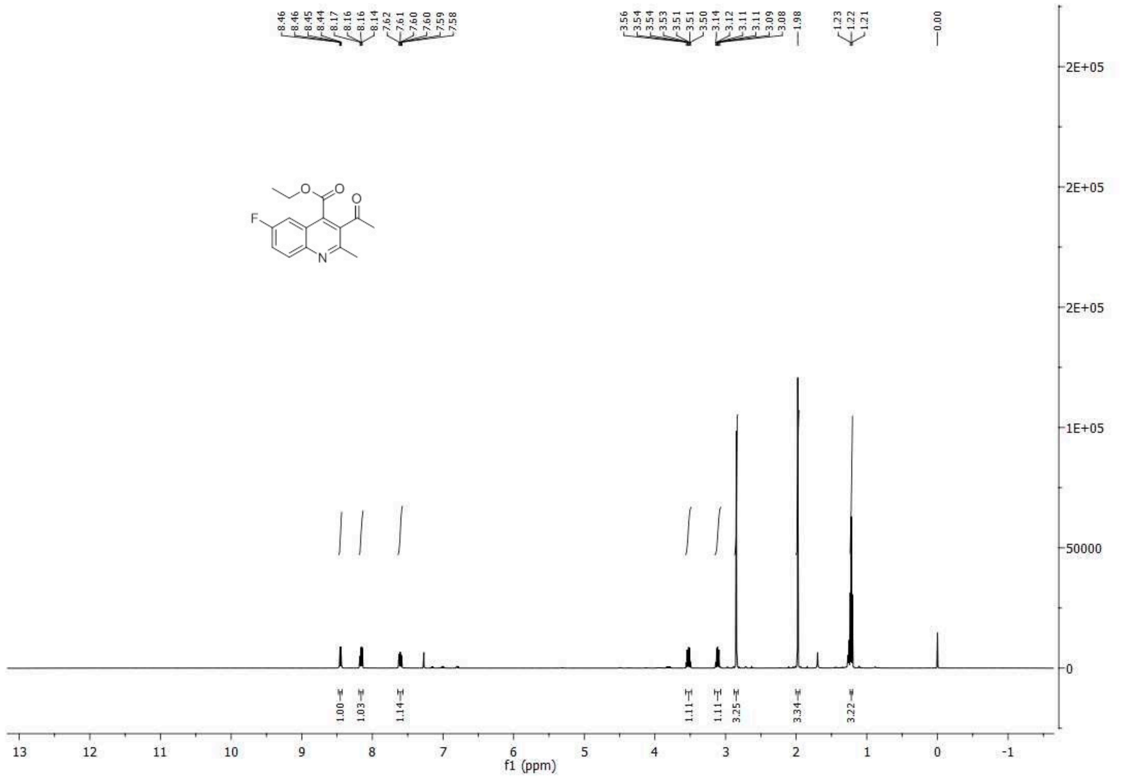

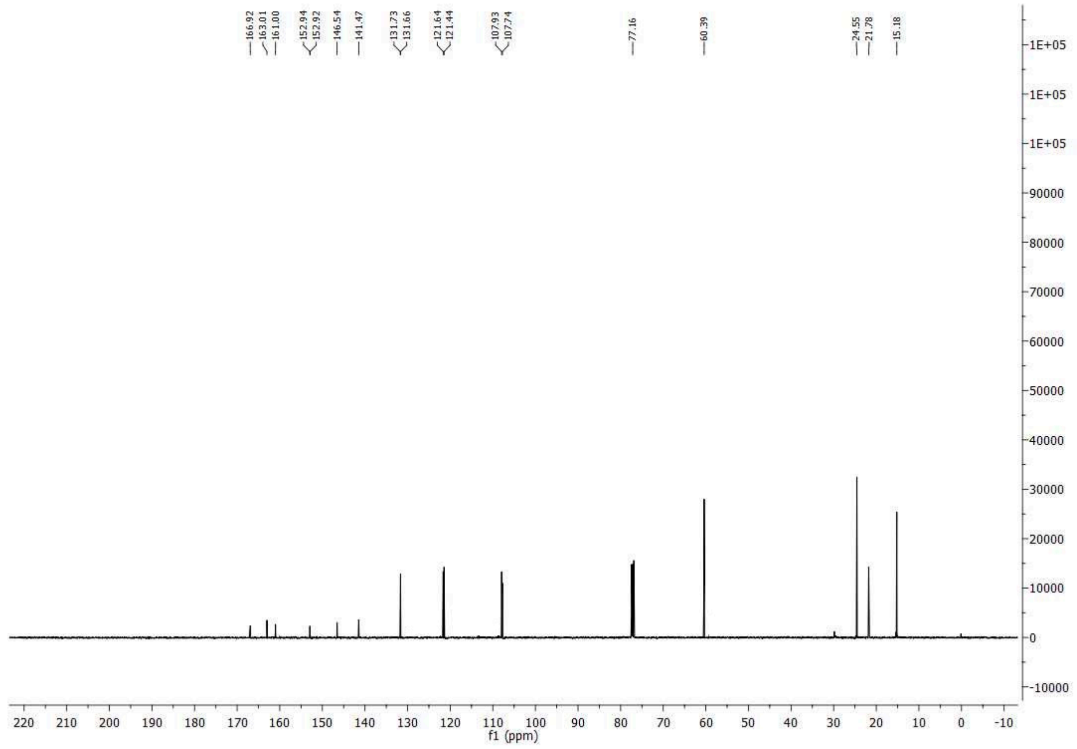

^{1}H and ^{13}C NMR of compound **4l**

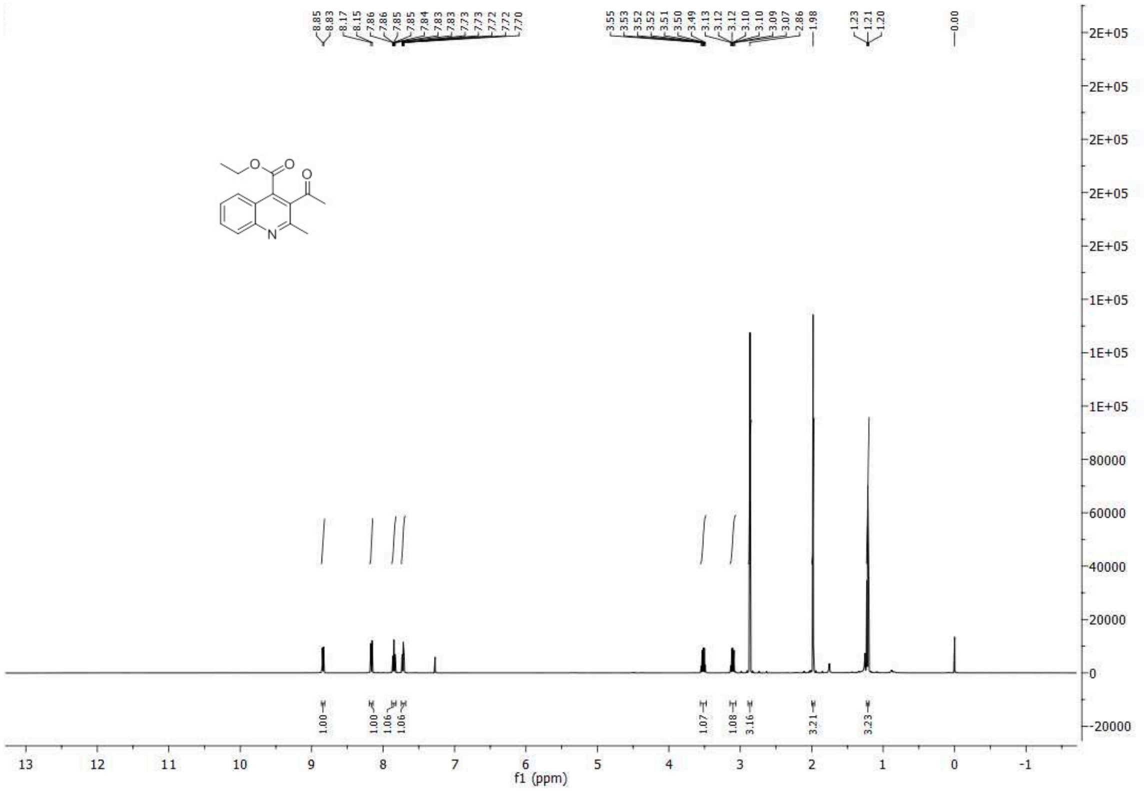

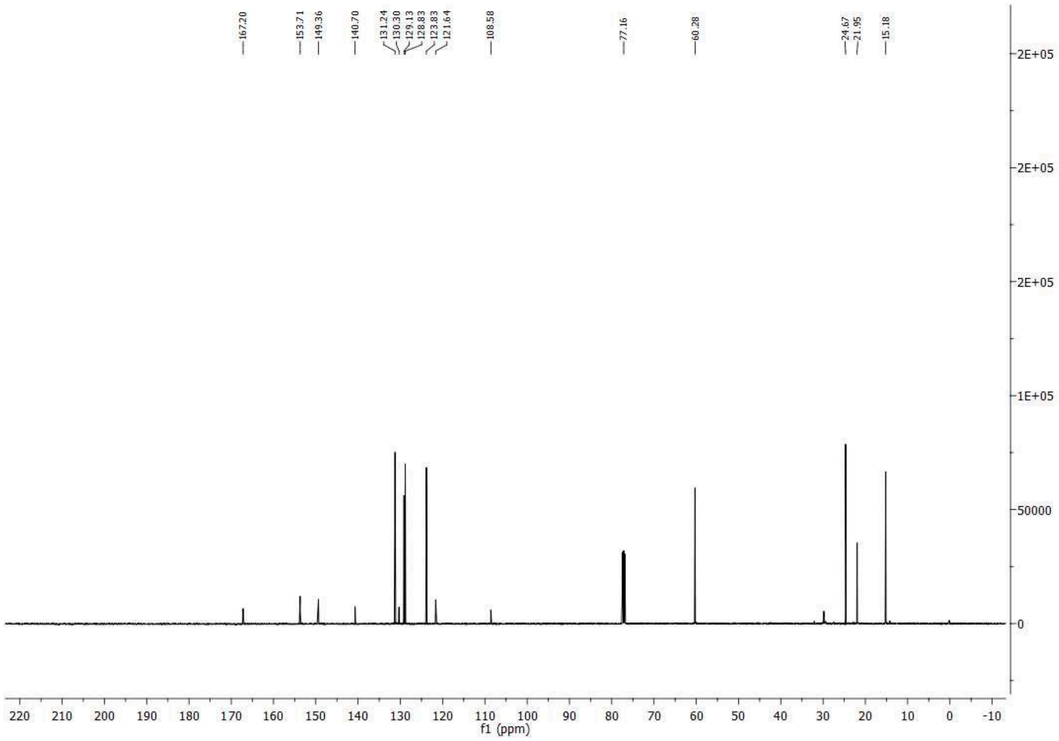

^{1}H and ^{13}C NMR of compound **4m**

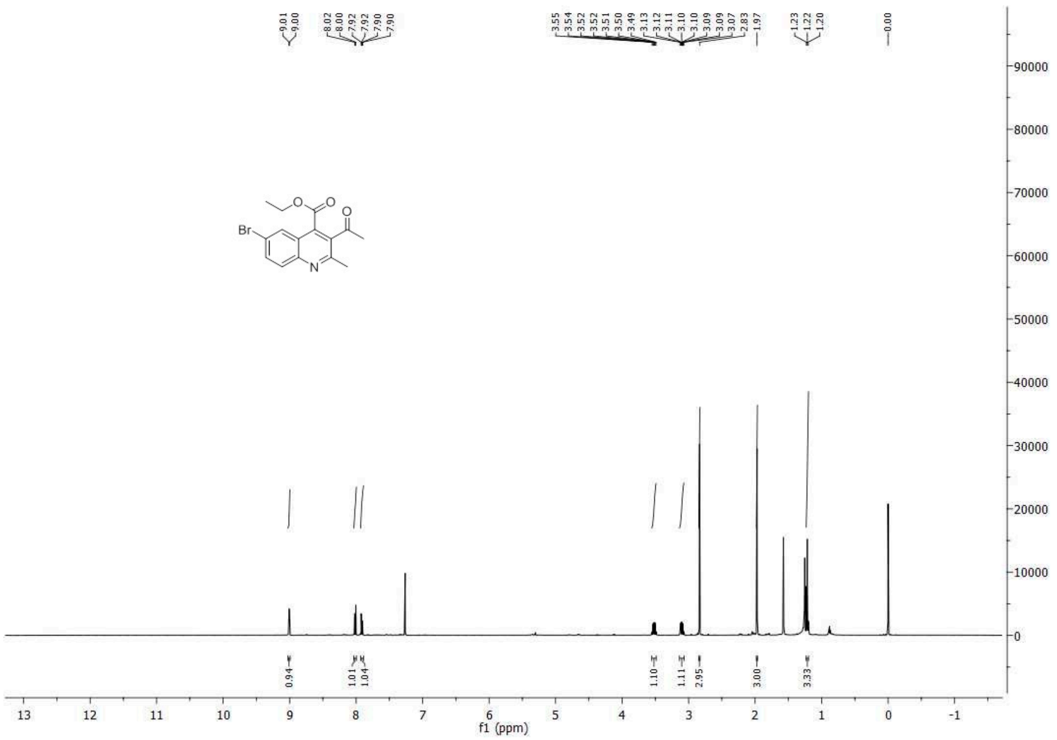

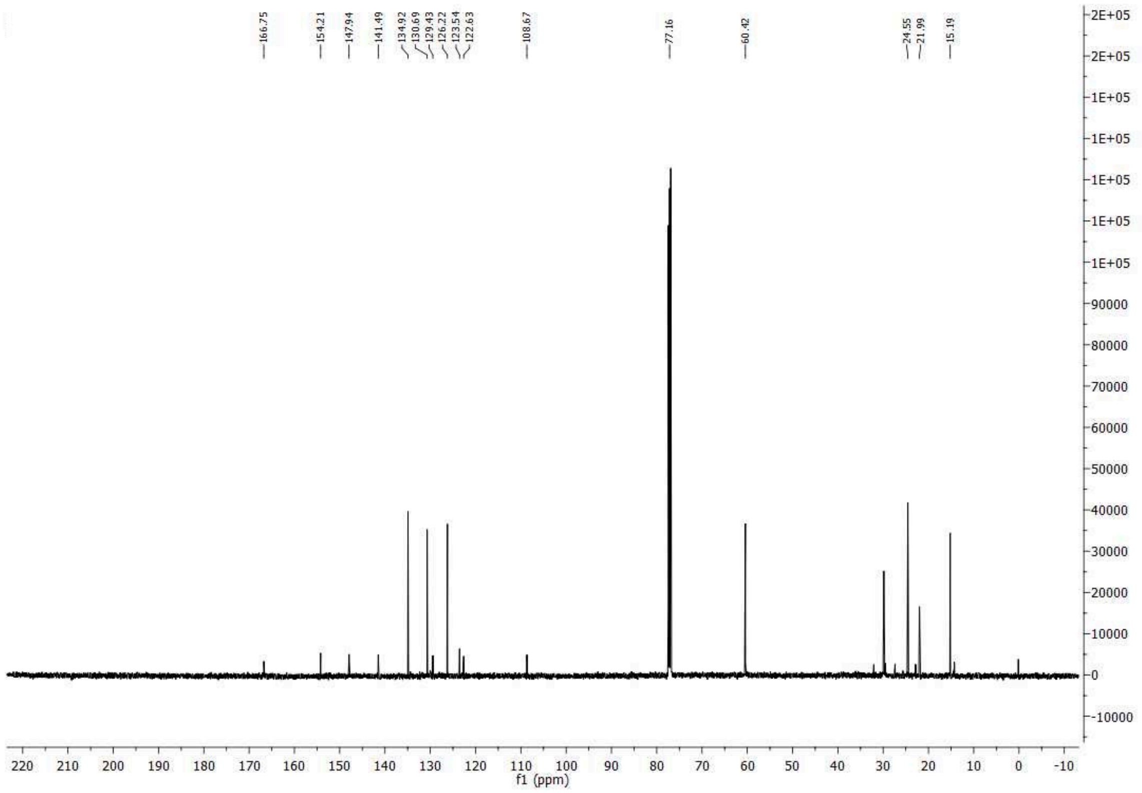

^{1}H and ^{13}C NMR of compound **4n**

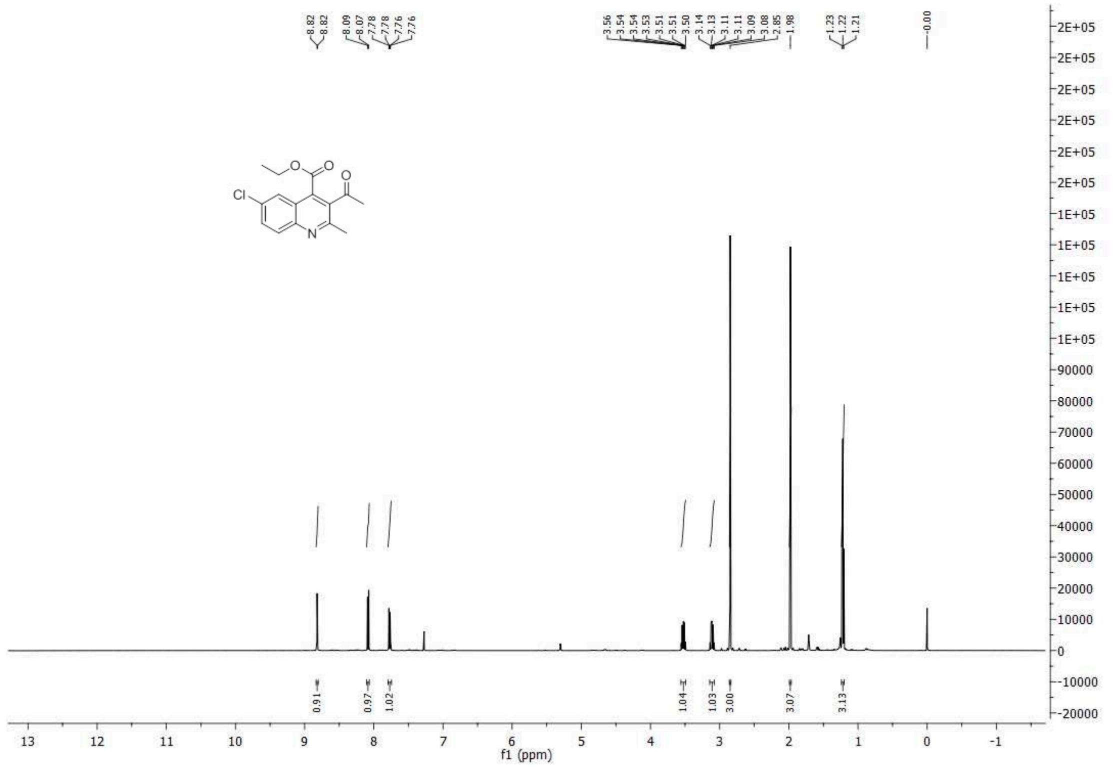

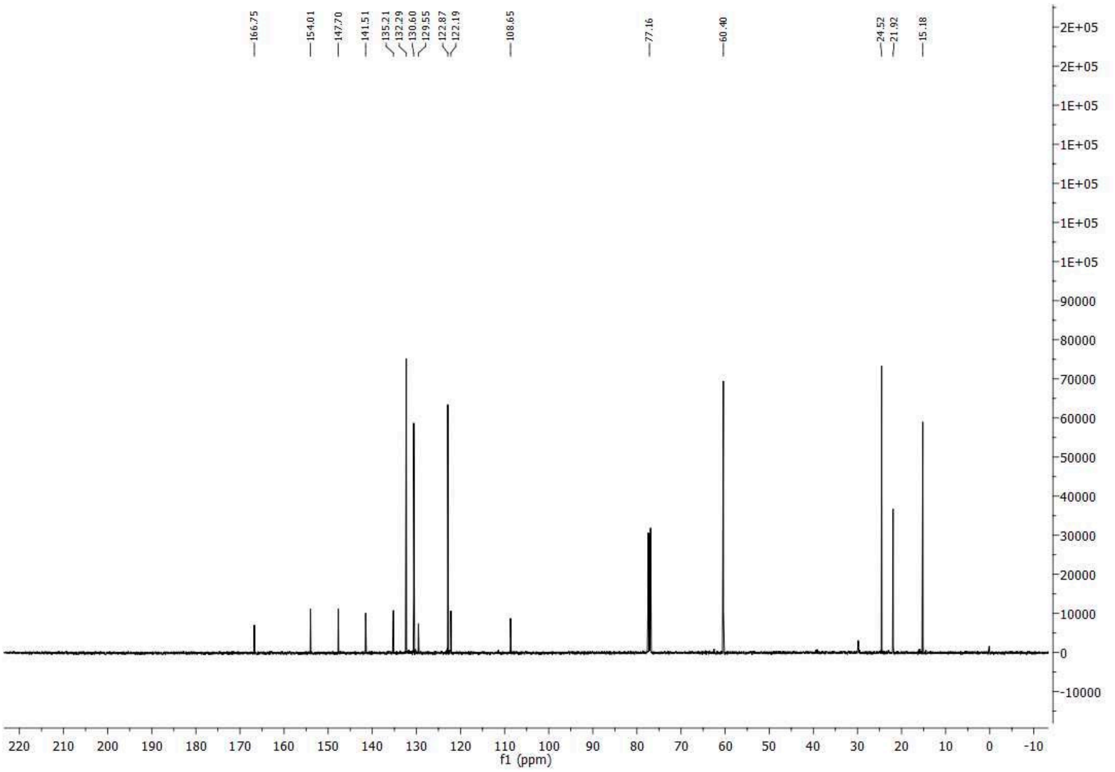

^{1}H and ^{13}C NMR of compound **4o**

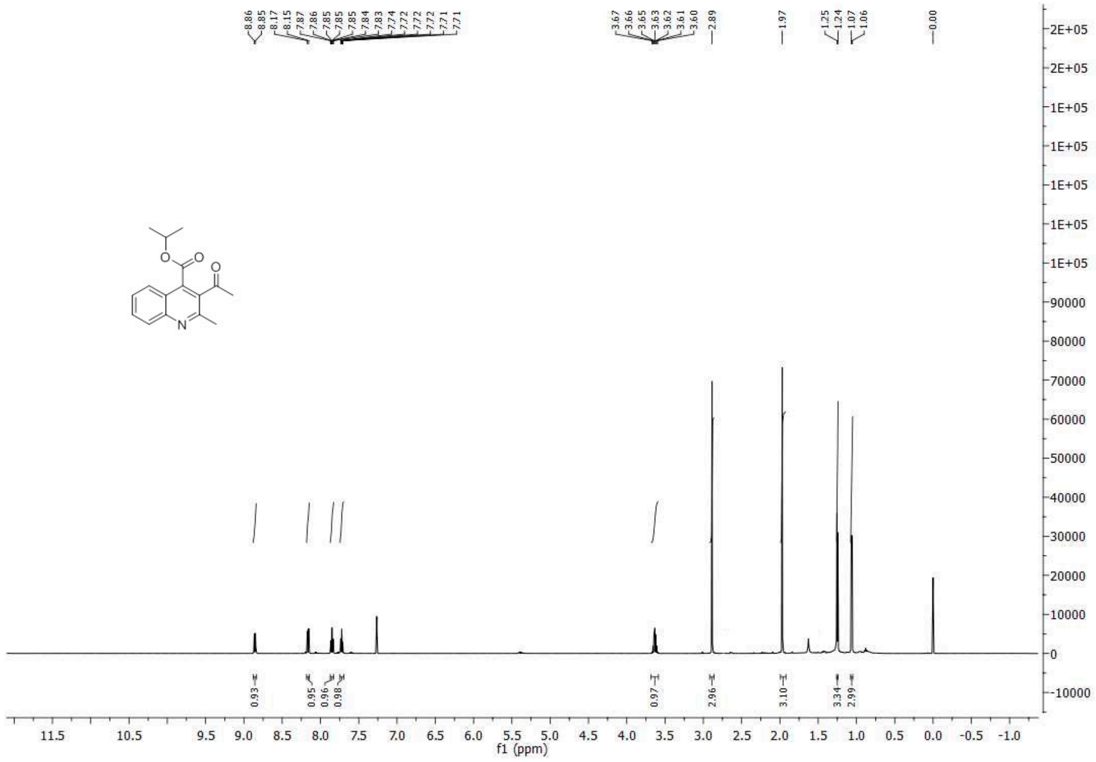

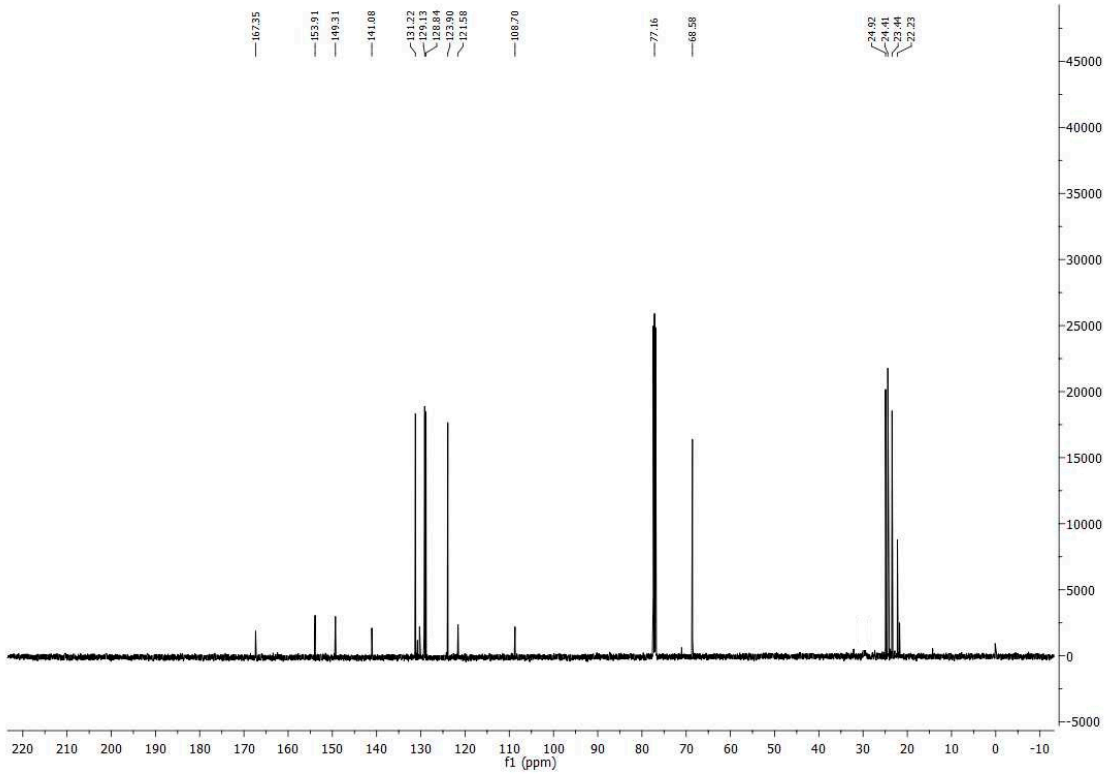

^{1}H and ^{13}C NMR of compound **4p**

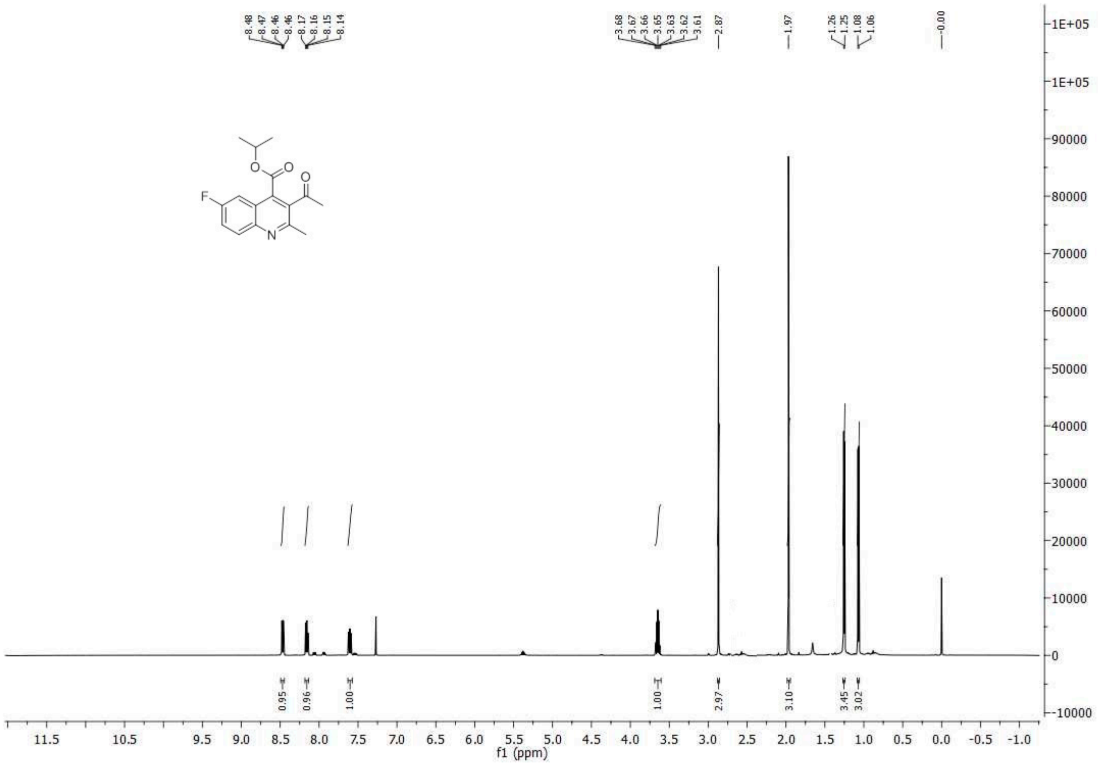

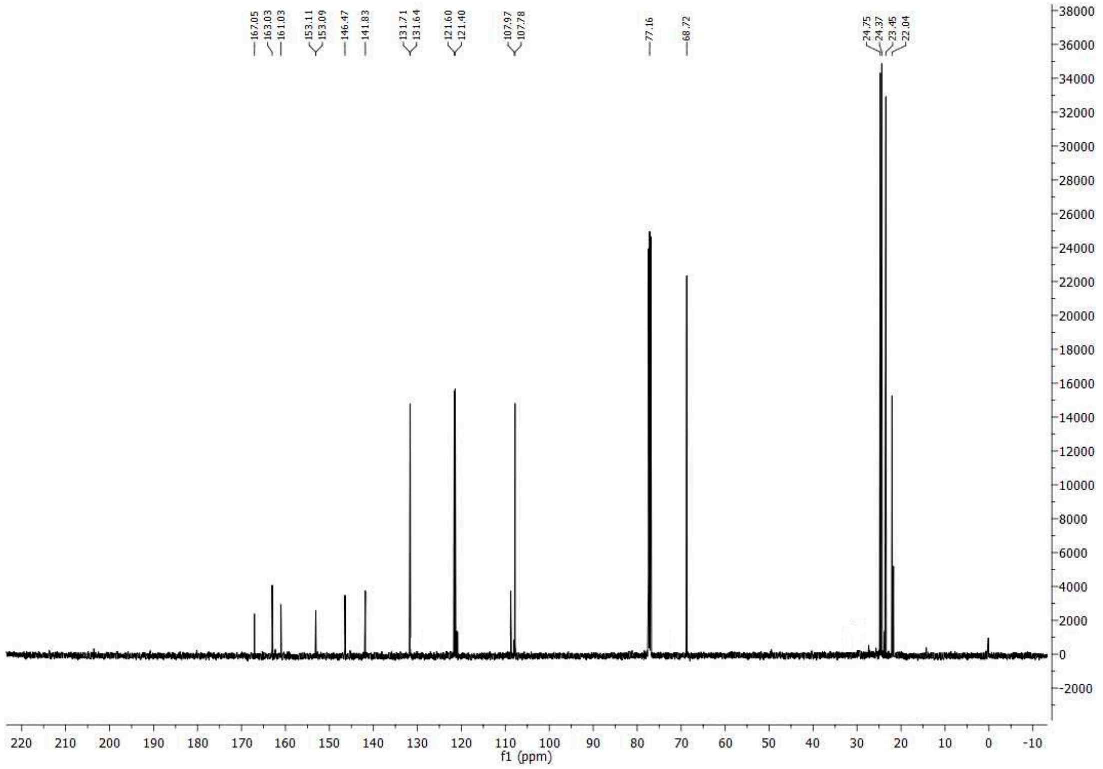

^{1}H and ^{13}C NMR of compound **4q**

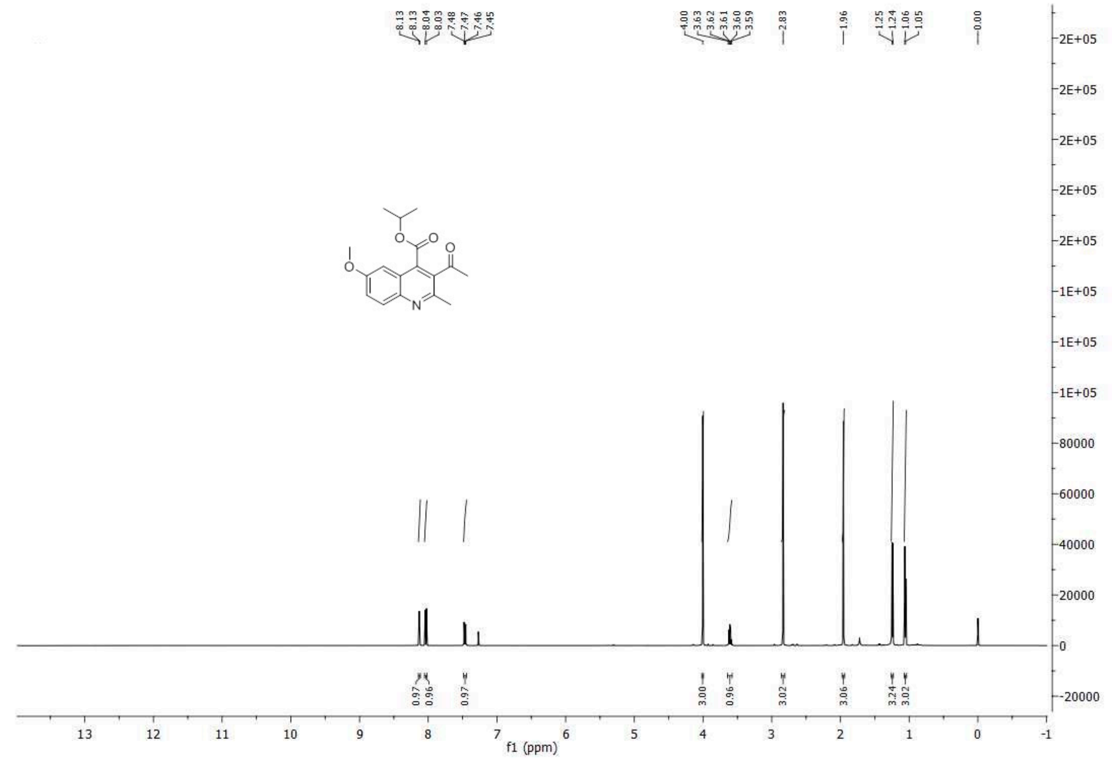

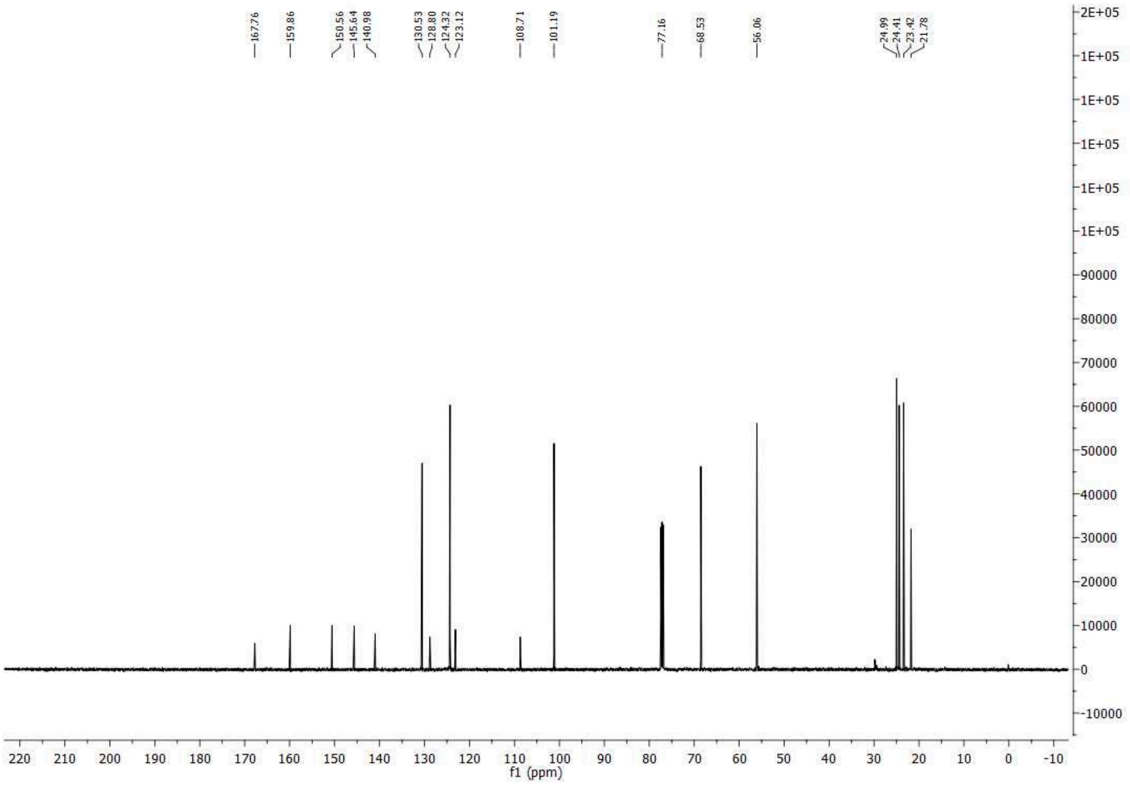

^{1}H and ^{13}C NMR of compound **4r**

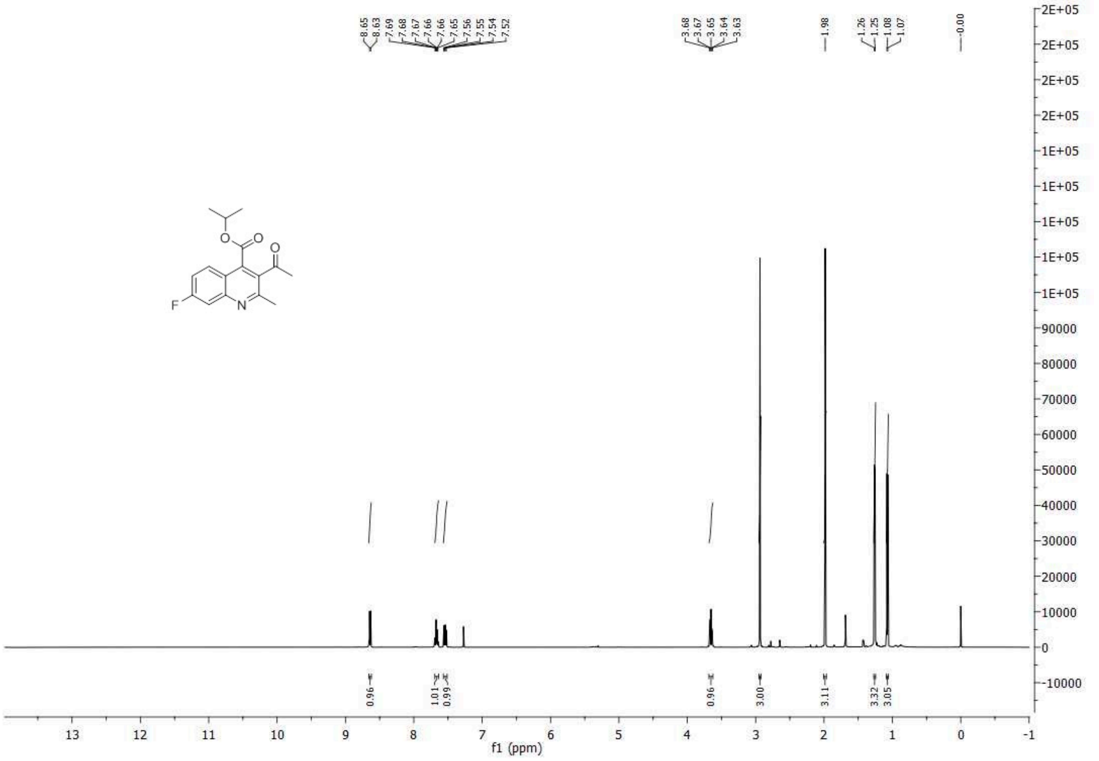

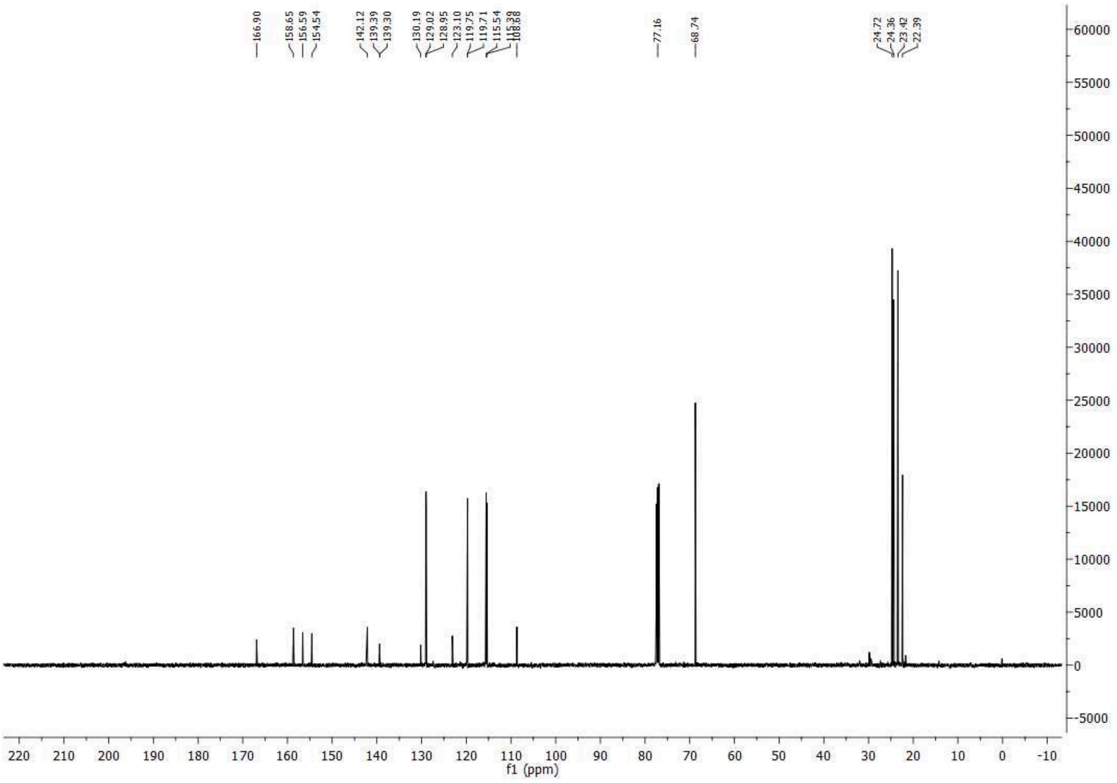

^{1}H and ^{13}C NMR of compound **4s**

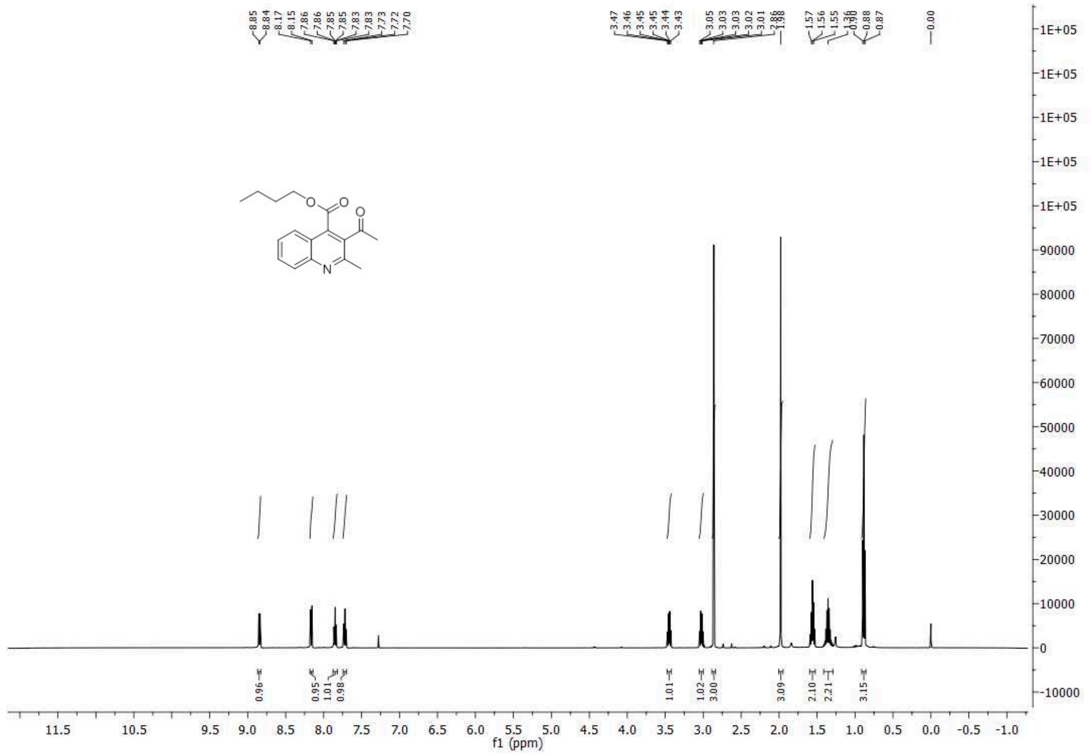

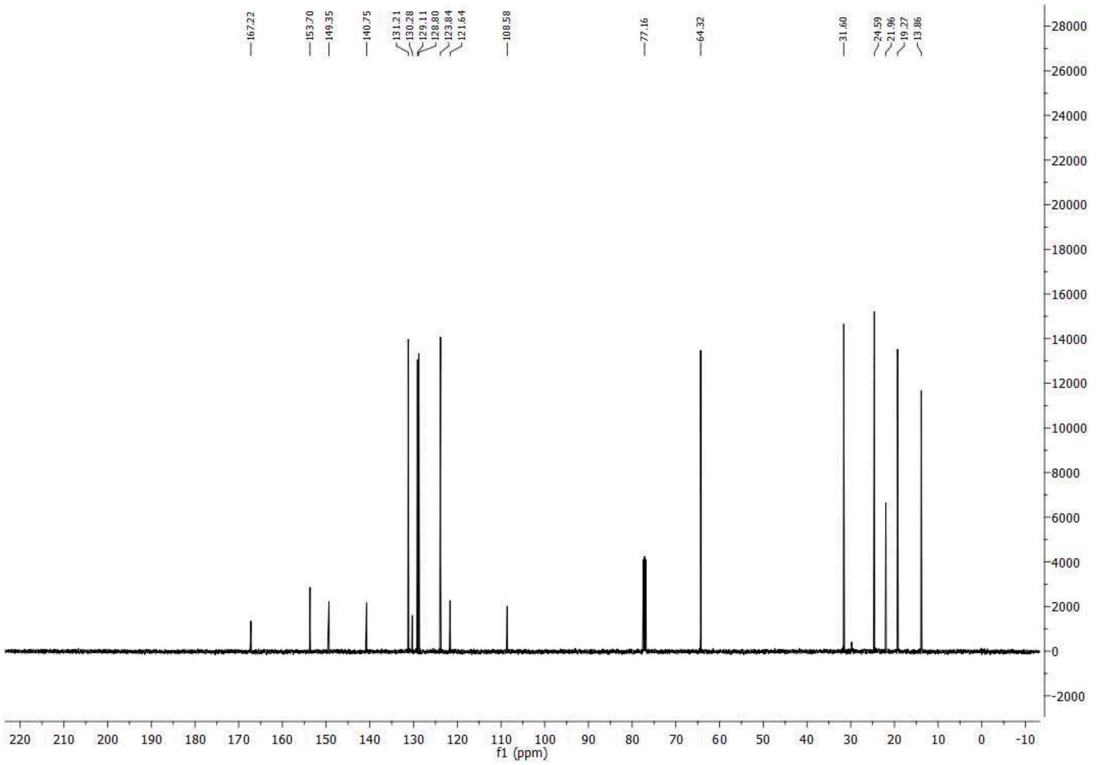

^{1}H and ^{13}C NMR of compound **4t**

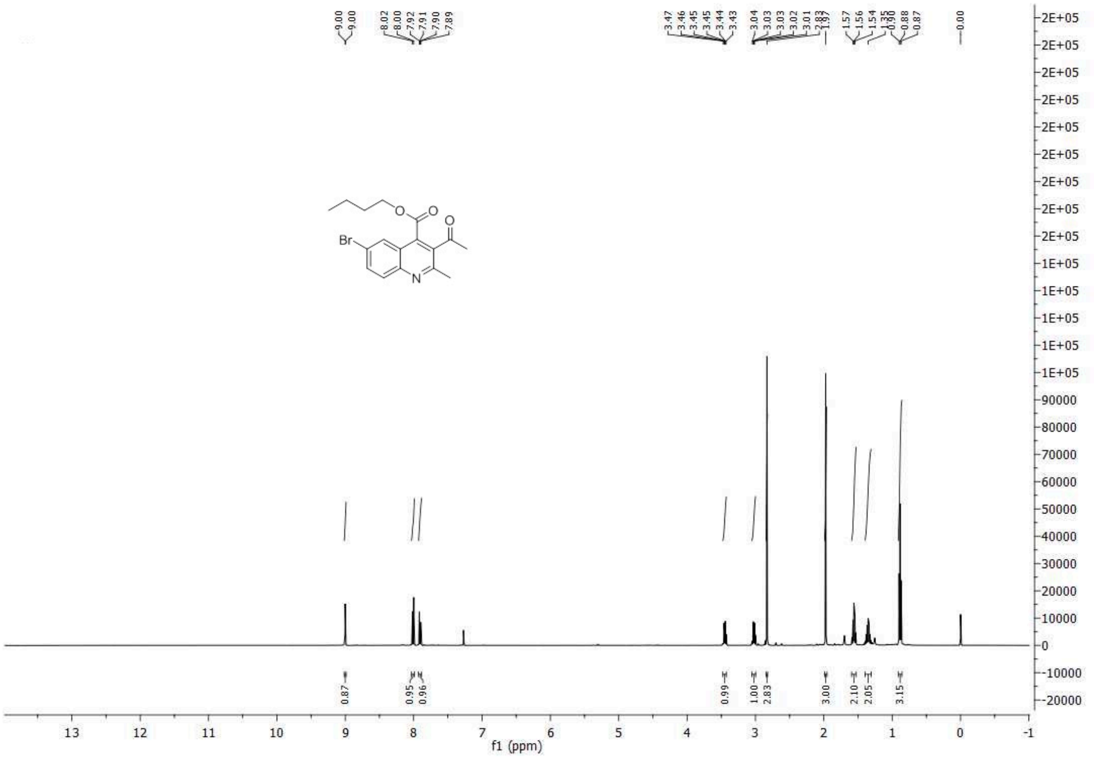

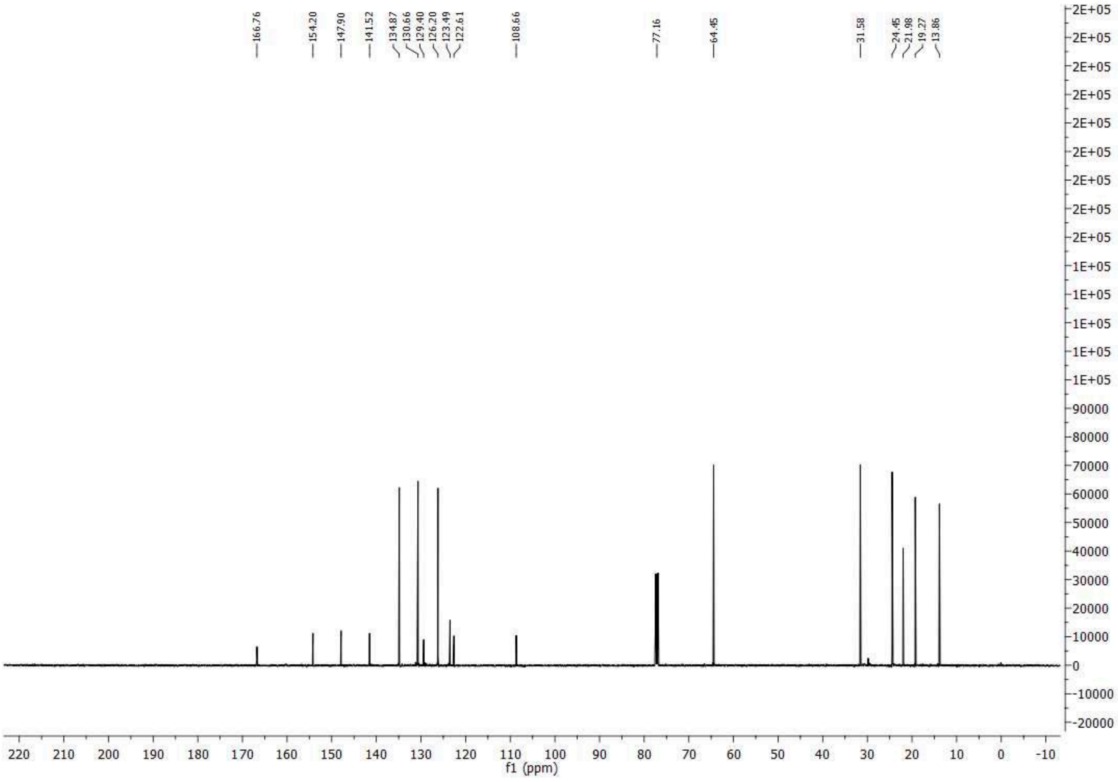

^{1}H and ^{13}C NMR of compound **4u**

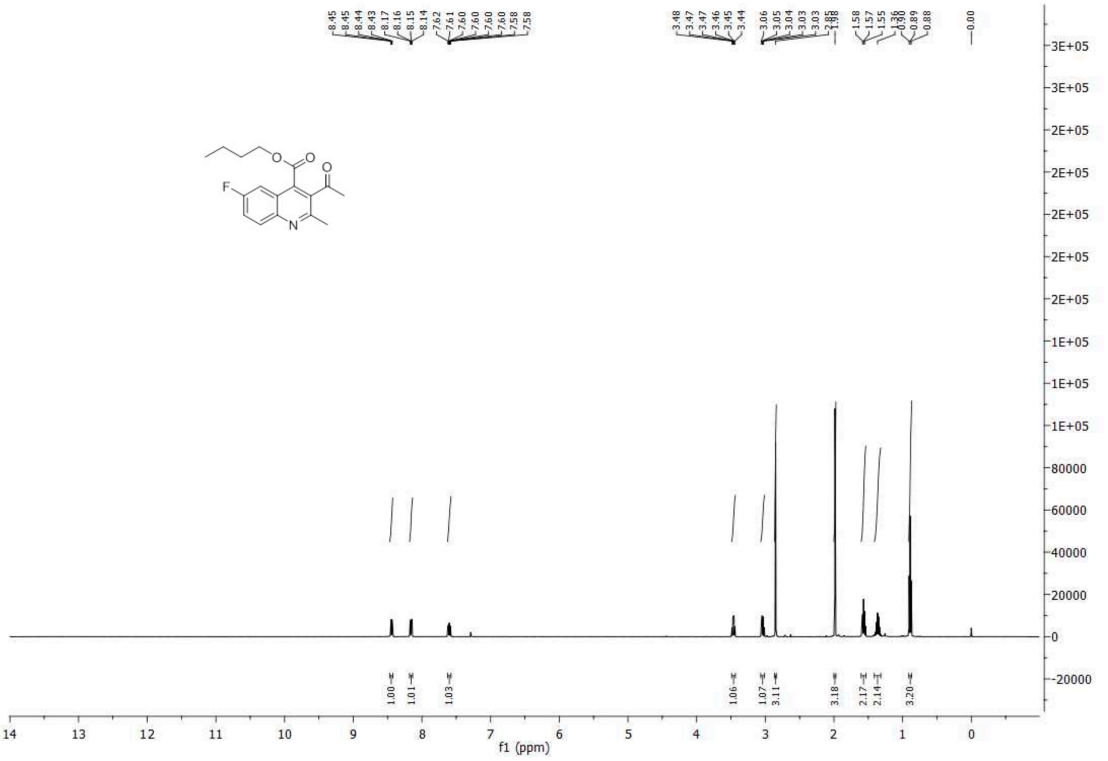

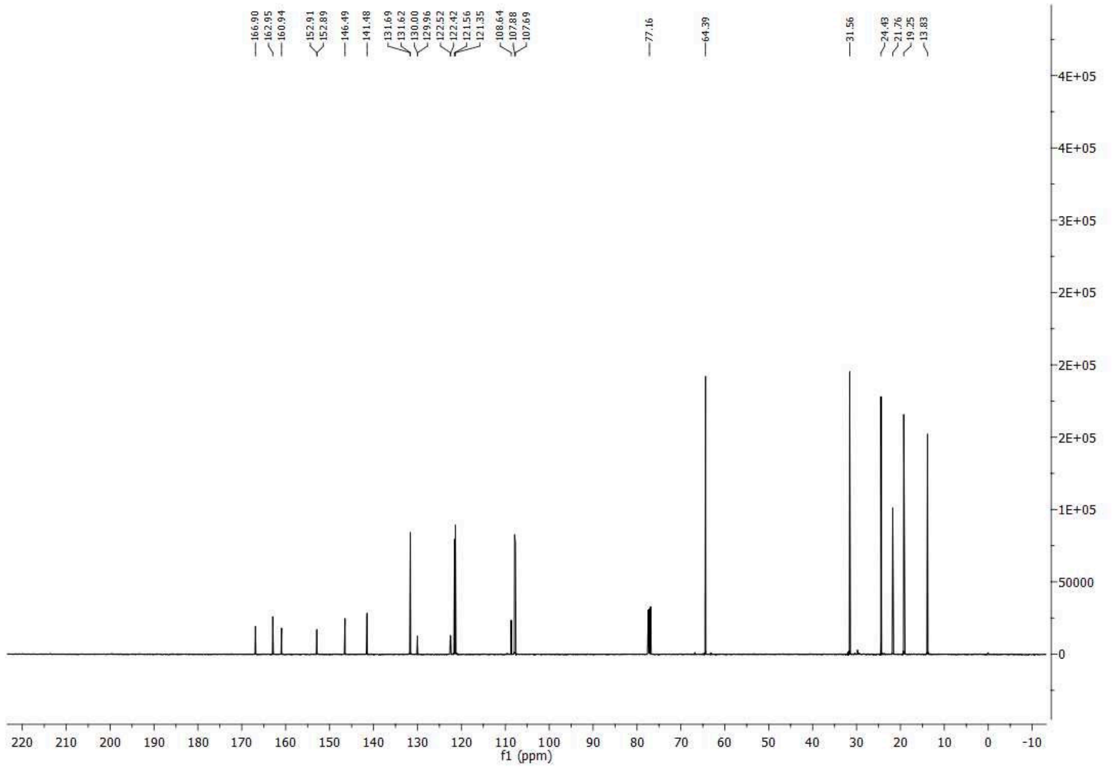

^{1}H and ^{13}C NMR of compound **4v**

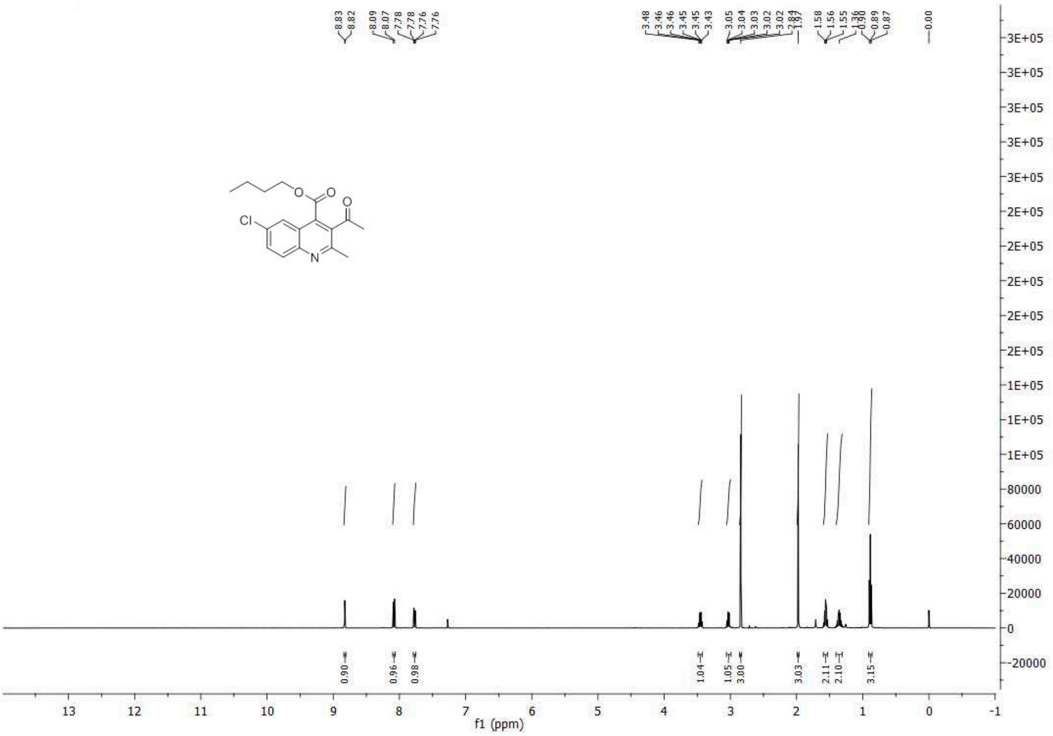

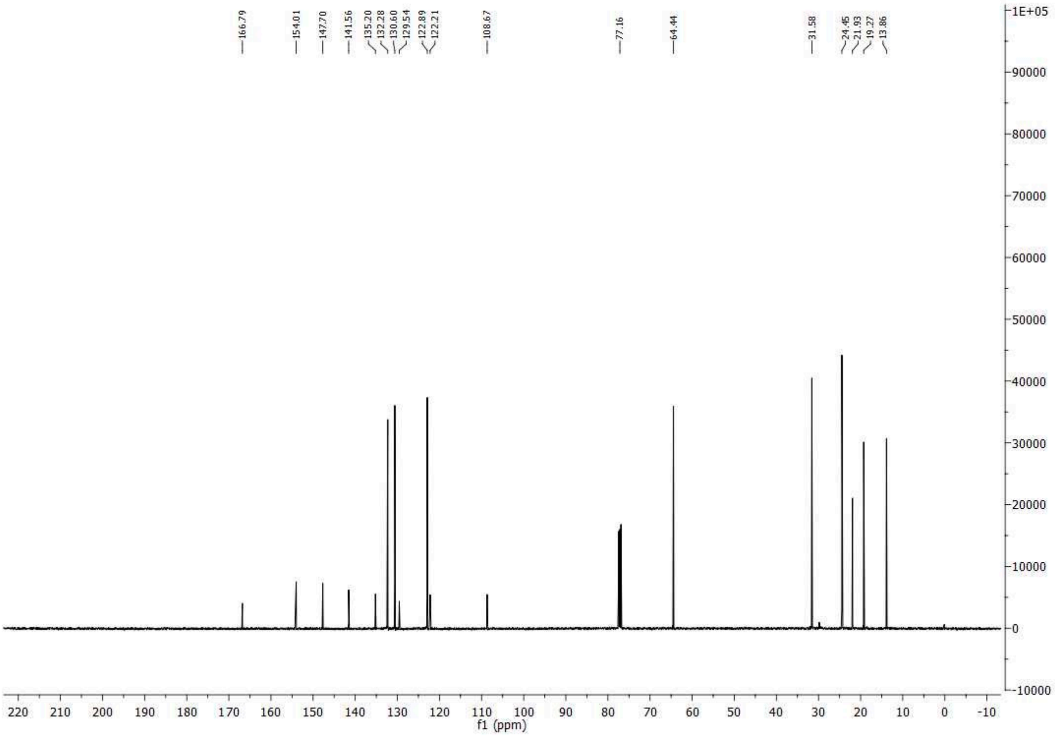

^{1}H and ^{13}C NMR of compound **4w**

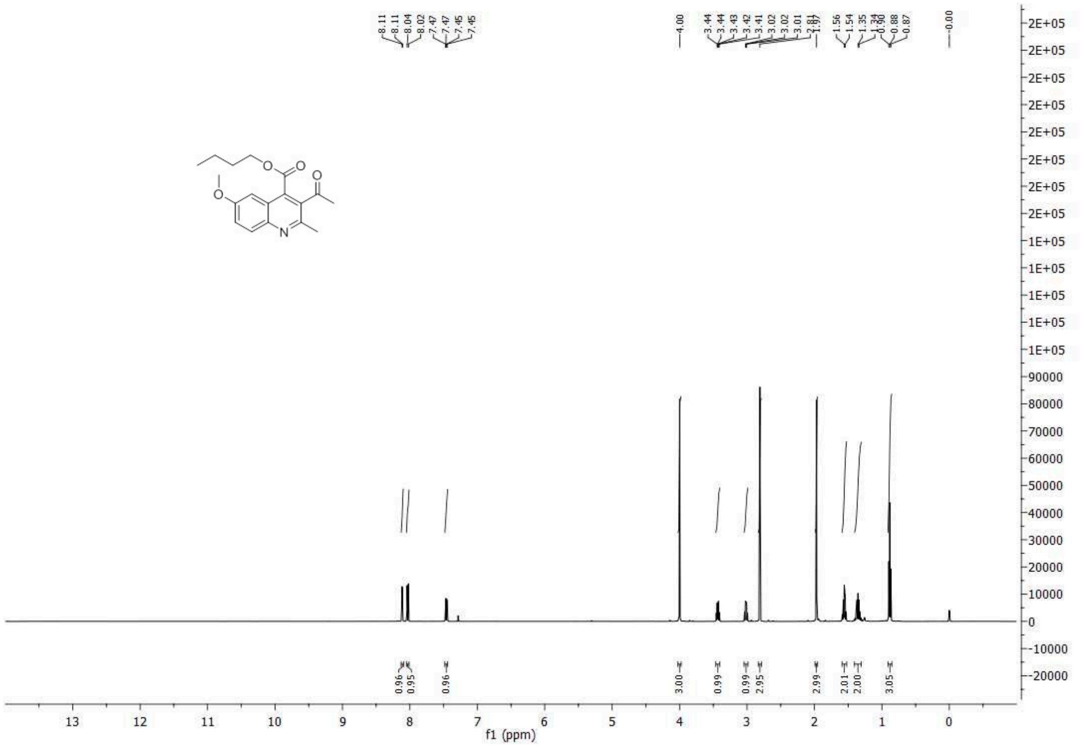

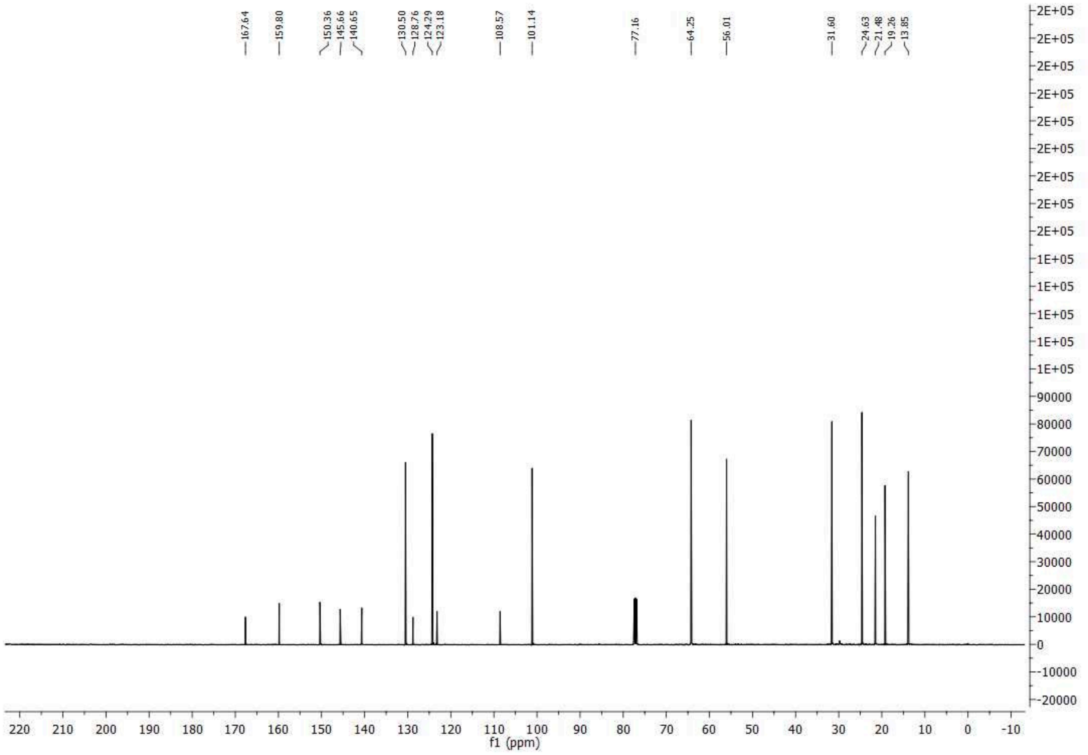

Supplement: Supplementary file 1 [file polymers-12-00393-s001.pdf]
